# Permeation of small extracellular vesicles across a human blood-brain barrier transwell model remains below particle detection limits, even under oxygen/glucose deprived conditions

**DOI:** 10.1080/21688370.2025.2610016

**Published:** 2026-01-09

**Authors:** Adrián Klepe, Sonja Damberger, Ana Špilak, Andreas Brachner, Winfried Neuhaus

**Affiliations:** a Competence Unit Molecular Diagnostics, Center for Health and Bioresources, AIT - Austrian Institute of Technology Gmbh Vienna, Austria; b Faculty of Medicine and Dentistry, Danube Private University Krems, Austria

**Keywords:** Blood-brain barrier, *in* *vitro* model, permeation, small extracellular vesicles, stroke, uptake

## Abstract

Ischemic stroke disrupts blood-brain barrier (BBB) integrity and alters small extracellular vesicle (sEV) signaling, yet the mechanisms underlying sEV transport across compromised barriers remain poorly understood. This study investigated BBB-sEV interactions under normal and stroke-mimicking oxygen/glucose deprivation (OGD) conditions using an *in vitro* human BBB co-culture model consisting of brain capillary endothelial (BCECs, hCMEC/D3) and astrocytoma cells (1321N1). Model characterization revealed that co-culture with 1321N1 cells enhanced BBB vulnerability to OGD compared to hCMEC/D3 mono-culture. OGD exposure (5 h and 24 h) progressively decreased transendothelial electrical resistance (TEER) and increased FITC-dextran 4 (FD4) permeability, with more severe impairment in co-cultures (e.g. TEER – after 5 h: 0.85-fold, after 24 h: 0.55-fold for co-culture related to mono-culture). Following 19 h oxygen and glucose restoration (‘recovery’) after 5 h OGD, barrier integrity loss was halted but not reversed. Transcriptomic analysis revealed adaptive cellular responses including upregulated glucose transporter 1 (GLUT1) and vascular endothelial growth factor (VEGF), alongside temporal changes in tight junction protein expression (CLDN5, CDLN6). sEV secretion kinetics in apical and basolateral compartments demonstrated that both cell types released particles in response to OGD in a time-dependent manner, with co-cultures showing enhanced secretion compared to mono-cultures. sEV uptake and permeation studies using eight cancer cell line-derived sEVs revealed cell-origin dependent internalization patterns by BCECs, with the highest uptake for HEK293T and SH-SY5Y sEVs. These internalized sEVs were predominantly targeted to lysosomes. Despite severe barrier disruption due to OGD transcellular permeation of single sEV particles was not detectable.

## 1. Introduction

Stroke represents one of the leading causes of death and long-term disability worldwide, imposing a substantial socioeconomic burden on healthcare systems.^1^ Ischemic stroke, accounting for approximately 87% of all stroke cases, occurs when blood flow to the brain is obstructed.^2^ This obstruction leads to a cascade of pathophysiological events, caused by shortened oxygen and glucose supply in the affected brain region, leading to inflammatory reactions, oxidative stress and further detrimental consequences, such as tissue necrosis.^3^

The blood-brain barrier (BBB), a specialized interface maintaining central nervous system (CNS) homeostasis, is first and directly affected during cerebral ischemia. Composed of brain capillary endothelial cells (BCECs) interconnected by tight junctions (TJs), the BBB facilitates and regulates the exchange of molecules between blood and the CNS. In contrast to peripheral capillaries, the endothelial cell layer of the BBB is characterized by a high transendothelial electrical resistance (TEER) which reflects the tight paracellular sealing preventing passive diffusion between the BCECs, and the expression of specialized influxand efflux transporters (many belonging to the ABC or SLC gene families) mediating the regulated transport of e.g. glucose, amino acids, and lipids. The high barrier tightness is furthermore supported by a low rate of pinocytosis and the absence of fenestrae.^4^ During stroke, BBB integrity is compromised, as TJ proteins such as occludin, claudin-5 and ZO-1, and ABC transporters such as P-glycoprotein (P-gp, encoded by ABCB1), breast cancer resistance protein (BCRP, encoded by ABCG2), and multidrug resistance-associated protein 1 (MRP1)^5–8^ are affected. BCECs respond to the lack of oxygen and glucose *inter alia* by an upregulation of the glucose transporter GLUT1 and activating vascular endothelial growth factor (VEGF) signaling.^9–11^

The BBB is embedded in the neurovascular unit (NVU) composed of several cell types, including pericytes which share the basal lamina with the endothelial cells; astrocytes covering the parenchymal surface of the brain capillaries with their end-feet processes and neurons which can modify BBB function.^12^ The microenvironment and cell-cell contacts within the NVU are essential for the function and maintenance of BBB tightness and ABC transporter activities.^13^ Co-cultivation with astrocytes, and/or glioma cells was reported to be necessary to achieve functional BBB breakdown in *in vitro* cerebral ischemia models within an *in vivo*-relevant time frame of 4–5 hours.^9,14,15^ Pericytes, the most adjacent cells to BCECs on the CNS side, maintain barrier integrity by regulating paracellular permeability and TJ expression profiles at the BBB, even under experimentally induced oxygen/glucose deprivation (OGD) conditions.^11,16–18^

Multiple studies have documented the uptake of small extracellular vesicles (sEVs) in *in vitro* BBB models under both physiological and pathological conditions.^19–23^ Small EVs represent cell-derived, membrane-enclosed transport vehicles for a variety of biological molecules such as RNA, DNA, proteins and lipids. Partially, this cargo reflects the cellular origin of the sEV, hence the cargo composition can be different between healthy and pathological states.^2^ The cargo-related changes in EVs have been demonstrated following ischemic stroke insults: elevated levels of tumor necrosis factor alpha (TNFα), interleukin 6 (IL-6) and VEGF,^24^ and the astrocyte marker excitatory amino acid transporter 1(EAAT1)^25^ were present in EVs isolated from post-ischemic plasma. Altogether, EVs can be potential carriers of biomarkers for disease diagnostics, and seem to play an active role in different phases of stroke as a vehicle for intercellular signaling.^26^

Human BCECs were shown to internalize sEVs from various cellular origins, including breast cancer,^21,23^ neutrophils,^19^ and melanoma.^20^ When the BCEC layer was compromised by treatment with LPS,^27^ cytokines^22,28–30^ or virion carrying EVs,^31^ an increased permeation of bioluminescently, radioactively or fluorescently labeled EVs across the endothelial barrier was observed, though, due the applied methodologies, it remained undetermined whether whole EVs or only labeled cargo transversed the BCEC layer.^27,28,31^

Most state-of-the-art *in vitro* models relevant for studying BBB dysfunction and disruption during cerebral ischemia, employ OGD.^32–36^ Various mono- and co-culture systems have been established, combining BCECs (primary cells, cell lines, or differentiated from induced pluripotent stem cells) and one or more cell types physiologically found within the NVU – most commonly astrocytes (primary or astrocytoma) and pericytes (primary), but also neuronal cells.^9,14,15,37,38^ While ischemic conditions alter EV production and secretion in BCECs^30,39,40^ and astrocytes,^41,42^ the specific effects of OGD on sEV release from hCMEC/D3 BCECs and 1321N1 astrocytoma cells in co-culture, have not been investigated in detail yet. Additionally, sEV uptake by BBB cells and permeation across the BBB during different phases of stroke remain to be elucidated in detail.

In the current study, OGD effects on BBB integrity and permeability were approached in an *in vitro* co-culture model comprising endothelial hCMEC/D3 and 1321N1 astrocytoma cells. BBB-related gene expression, sEV release, and the uptake and permeability of sEVs originating from eight different cell types were investigated under normal conditions and the two selected ones under mimicked cerebral ischemia.

## 2. Materials and methods

### 2.1 Cell culture and authentication

All cell lines used in this study were authenticated by SNP genotyping (Eurofins Genomics). Cell lines for sEV purification had been established and described previously: HEK293T, human embryonic kidney cells^43^; 1321N1, human astrocytoma cell line^44^; A549, lung adenocarcinoma^45^; MO3.13, human oligodendrocytes^46^; MDA-MB-231, breast adenocarcinoma^47^; SK-MEL-28, skin malignant melanoma^48^; SH-SY5Y, neuroblastoma^49^; HMC3, human microglia cell line^50^ (Figure S1).

HEK293T, 1321N1, A549, MO3.13 and MDA-MB-231 were maintained in Dulbecco’s Modified Eagle Medium (DMEM; #D5796; Sigma-Aldrich, St. Louis, MO, USA) supplemented with 10% fetal bovine serum (FBS; #F9665; Sigma-Aldrich) and 100 U/mL Penicillin, 100 µg/mL Streptomycin (1% P/S; #A2213; Merck, Darmstadt, Germany) (DMEM++) at 37°C in a humidified atmosphere (95%) with 95% air and 5% CO_2_. Cells were detached at 80–100% confluency with 0.25% trypsin/EDTA (#P10-02310; PAN-Biotech, Aidenbach, Germany) once or twice per week with subcultivation ratios as follows: 1:6–1:10 for HEK293T, 1:8–1:10 for 1321N1, 1:6–1:8 for A549 and for MO3.13, and 1:4–1:6 for MDA-MB-231.

SK-MEL-28 were cultivated in RPMI-1640 (#R8758; Sigma-Aldrich) supplemented with 10% FBS and 1% P/S, and split every 3–4 days at approximately 90% confluency in a ratio of 1:6–1:8. SH-SY5Y cells were maintained in DMEM/F12 (1:1) (#21331046; Gibco^TM^ – Thermo Fisher Scientific, Waltham, MA, USA) supplemented with 10% FBS, 2 mM L-Glutamine (#G5713, Sigma-Aldrich) and 1% P/S, and were split at approximately 90% confluency once or twice a week in a ratio of 1:10. The HMC3 cell line was grown in Eagle’s Minimum Essential Medium (EMEM; #M5650; Sigma-Aldrich) supplemented with 10% FBS, 2 mM L-Glutamine, 1 mM sodium pyruvate (#S9636; Sigma-Aldrich), 1x MEM Non-Essential Amino Acids Solution (MEM NEAA; #11140050; Gibco^TM^) and 1% P/S. Microglia were trypsinized once per week at 90–100% confluency and split at 1:3–1:5 ratio.

The human immortalized cell line hCMEC/D3 ^51^ was cultured as described previously.^9^ In brief, cells were grown on 0.5% gelatine-coated culture flasks (#22.151.02; SERVA Electrophoresis GmbH, Heidelberg, Germany) in EBM-2 endothelial basal media (#CC3156; Lonza, Basel, Switzerland) supplemented with 5% FBS, 1% P/S, 10 mM HEPES (#H0887; Sigma-Aldrich), 5 µg/mL ascorbic acid (#A4544; Sigma-Aldrich; stock solution: 1 mg/mL ascorbic acid in EBM-2 medium) and 1 ng/mL human basic fibroblast growth factor (hbFGF; #F0291; Sigma-Aldrich; stock solution: 200 ng/mL hbFGF in DPBS containing 0.1% BSA). hCMEC/D3 cells were detached with 0.25% trypsin/EDTA and seeded at a ratio of 1:3 once a week.

### 2.3 Small EV purification and labeling

sEVs were purified following established protocols.^22,52^ Briefly, cell lines were cultivated in 145 mm cell culture dishes (PD145; #639160; Greiner Bio-One, Kremsmünster, Austria) until they reached 70–80% density. After one wash with serum-free collection medium, cells were further incubated in sEV collection medium for 24 h (HEK293T) or 48 h (all other cell lines).

After collecting cell culture supernatants, floating cells, cell debris, apoptotic bodies, and larger vesicles were depleted by a three-step differential centrifugation at 4°C: 10 min at 500 × g, 15 min at 2000 × g and 25 min at 10,000 × g. Cleared media were concentrated to 1 mL retentate at 4000 × g using Amicon ultrafiltration units with 100 kDa cutoff (#UFC810096; Sigma-Aldrich). Small EVs were then purified from the retentate by size exclusion chromatography (SEC) using EX04 Exo-Spin midi columns (#EX04-20; Maxanim, Gentaur, Aachen, Germany). Fractions 7–12 were collected in DPBS in Protein LoBind® Tubes (#022431081, Sigma-Aldrich), then concentrated at 10,000 × g using 100 kDa Amicon Ultra 0.5 centrifugal tubes (#UFC510096; Sigma-Aldrich) until a final volume of 100–200 µL. Retentate volume was collected by inverting the inner filter and centrifuging at 1000 × g for 2 min. Before freezing and storing sEVs at −80°C, size, number, and surface charge of sEVs were determined by Nanoparticle Tracking Analysis (NTA) using a ZetaView® Quatt device (Particle Metrix GmbH, Inning am Ammersee, Germany). Serum-free media were incubated in PD145 dishes without cells and underwent the exact same purification process as cell-derived sEVs and served as negative controls (“media controls”) in all experiments.

The lyophilized CellTracker^TM^ Orange dye (CTO, #C34551; Invitrogen) was dissolved in DMSO to a stock solution of 10 mM, which was further diluted in DPBS to a 20 µM working solution. One part of 20 µM CTO dye solution was mixed with one part of sEV suspension in DPBS to obtain a final concentration of 10 µM CTO, which was then incubated light-protected, with gentle shaking, for 3 h at 37°C. After labeling, residual free dye was removed by adding twice a 20-fold volume of DPBS and purification via ultrafiltration with 100 kDa Amicon-Ultra 0.5 centrifugal filter tubes as described above. The same procedure was performed in parallel for media controls.

sEV labeling with PKH26 using the Red Fluorescent Cell Linker Kit (#PKH26; Sigma-Aldrich) was conducted according to the manufacturer’s instructions. Briefly, equal volumes of sEVs (or their media controls) and 20 µM PKH26 dye diluted in Diluent C were incubated for 1 h at 25°C. Unbound dye was removed with MicroSpin G-25 columns (#27532501; Cytiva, Marlborough, MA, USA). Labeled sEVs were eluted through the column by centrifugation at 750 × g for 2 min. For both sEV labeling methods, particle concentration, size distribution, and labeling efficiency were determined by NTA.

### 2.4 Nanoparticle tracking analysis

The hydrodynamic diameter, size distribution and concentration, zeta potential and fluorescent label of sEVs were measured and analyzed using a ZetaView® Quatt device and the software ZetaView® 8.05.12 SP2. The NTA device was calibrated routinely with PS 100 nm standard calibration beads (#110–0020; Particle Metrix; Inning Am Ammersee, Germany) according to the manufacturer’s instructions. All samples were diluted in Milli-Q water to achieve a concentration of 50–200 particles per field of view, ensuring reliable measurements as recommended by the manufacturer. For each scatter (S-NTA) and fluorescence mode (F-NTA) measurement, one cycle was performed by scanning 11 cell positions and capturing 30 (scatter) or 15 frames (fluorescence) per second.

In scatter mode, a sensitivity value of 75, shutter value of 100 and minimum particle brightness of 30 were set for measurement and analysis respectively. CTO- and PKH26-labeled samples were excited with a 520 nm laser and emission was detected with a 550 nm long pass filter at sensitivity value set to 92 and shutter value of 95, with activated “low bleaching” option. A minimum particle brightness of 25 was used for fluorescence analyses. Values are shown as the mean number of particles. Due to high background of media supplemented with BSA, the shutter had to be raised to 250 in scatter mode, and 100 in fluorescence mode when BSA was present. All measurements were done at 25°C as set in the instruments SOP.

### 2.5 Western blotting

Western blotting was conducted with minor modifications as published previously.^15,22^ Briefly, after harvesting media for sEV purification, cells were washed with DPBS and detached with a cell scraper (#179693, Thermo Scientific NuncTM) in 10 mL ice-cold DPBS, collected and centrifuged at 500 × g for 5 min at 4°C. The supernatant was aspirated the cell pellet was resuspended in 1 mL ice-cold DPBS and transferred into a Protein LoBind® tube. The cells were washed twice with ice-cold DPBS, followed by lysis in 900 µL high salt RIPA++ (hsRIPA++; 50 mM Tris-HCl pH 7.4 (#5429.3; Carl Roth), 500 mM NaCl (#0241; VWR International, Vienna, Austria), 150 mM EDTA (#E1644; Sigma-Aldrich), 1 mM Triton™ X-100 (#T8787, Sigma-Aldrich), 0.01% SDS (10% SDS solution, #A0676,1000; PanReac AppliChem, Darmstadt, Germany) supplemented with protease inhibitor cocktail (Roche cOmplete ULTRA tablets mini, #05892970001; Sigma-Aldrich) and phosphatase inhibitors (Roche PhosphoSTOP, #04906837001; Sigma-Aldrich) per PD145 dish. Lysates were placed on ice for 5 min before sonicating with a tip sonicator (Fisherbrand™ Q505 sonicator; Thermo Fisher Scientific; settings: 30% amplitude, 3 pulses, 1 sec per pulse), and stored at -80°C until further use. Protein concentrations of cell lysates were determined using the Pierce BCA Protein Assay kit (#23225; Thermo Fisher Scientific). For purified sEV samples, an aliquot was used to calculate the protein concentration by micro-BCA Protein Assay kit (#23235; Thermo Fisher Scientific) as described previously.^22^

Prior to sample loading, Laemmli buffer was added to the lysates, and proteins were denatured for 5 min at 95°C. 10–15 µg of total protein was loaded per lane of 10% SDS-polyacrylamide gels, and gel electrophoresis was performed at 130 V. Proteins were transferred onto polyvinylidene difluoride membranes (#162–0177; Bio-Rad Laboratories, Hercules, CA, USA) using a tank blotter (Mini Protean; Bio-Rad) at 40 mA constant current per each membrane overnight at 4°C. Membrane blocking, incubations with primary and secondary antibodies and signal detection were carried out as described previously.^15^ The following primary antibodies were applied (1:1000): anti-Alix (#2171S; Cell Signaling Technology), anti-CD81 (#66866–1-Ig; ProteinTech GmbH), anti-CD9 (#13403S; Cell Signaling Technology), TSG101 (#67381–1-Ig; ProteinTech GmbH), GM130 (#11308–1-AP; ProteinTech GmbH). Two secondary antibodies were subsequently applied to the corresponding primary antibodies (1:5000): anti-mouse-IgG (#7076; Cell Signaling Technology) and anti-rabbit-IgG (#LNA934V/AH; GE Healthcare UK Limited). The HRP-coupled anti-β-actin (#A3854; Sigma-Aldrich) was diluted to 1:20,000.

### 2.6 In vitro BBB model and OGD conditions

All transwell insert experiments utilized the optimized experimental system for sEV studies as described before,^22^ applying 1.0 µm pore-sized Greiner ThinCert® membrane inserts (#662610; Greiner Bio-One) in 24-well plate formats. ThinCerts® were coated by incubating the membranes apically with a mixture of 4 µg of collagen IV and 10 µg of fibronectin in DPBS per insert membrane (#C5533; #F1141; Sigma-Aldrich; 1 mg/mL collagen IV stock: 1 mg/mL in 0.005% acetic acid) overnight at 37°C. After incubation with the coating mixes, inserts were washed twice with sterile DPBS and immediately used for experiments.

For screening experiments, hCMEC/D3 cells were seeded onto coated, 1.0 µm pore-sized Greiner 24-well ThinCert® (as described above) at a density of 40,000 cells/cm^2^. Medium was changed on days 2 and 5 after seeding; on day 5 FBS in the medium was reduced to 0.25%. For experiments on day 6, 10^9^ CTO-labeled sEVs per mL or the equivalent volume of medium control in DPBS was added to the donor compartment – either apically or basolaterally, in EBM-2 medium with 1% P/S. For consistency, 3.5% DPBS as the final concentration was applied. On the receiver compartment, EBM-2 medium supplemented with 1% P/S and 1% bovine serum albumin (BSA; #A7096; Sigma-Aldrich) was used. Each experiment was repeated three times independently, with two to four replicates for each condition per experiment.

For uptake inhibition studies, hCMEC/D3 cells were seeded into collagen IV/fibronectin coated 24-well plate wells at a density of 40,000 cells/cm^2^. Medium change on days 2 and 5 was performed in the same manner as for screening experiments. For experiments on day 6, cells were pretreated with the following endocytosis inhibitors at selected concentrations for 30 min: chlorpromazine (CPZ, 28 µM; #C8138-5G; Sigma-Aldrich), nystatin (NYS, 25 µM; #475,914-1 GM; Sigma-Aldrich), filipin III (FIL, 1.5 µM; #SAE0087; Sigma-Aldrich), cytochalasin D (CCD, 5 µM; #F9765-25 MG; Sigma-Aldrich), chloroquine (CLQ, 30 µM; #C6628-50G; Sigma-Aldrich), or DMSO as a vehicle control (#A994.2; Carl Roth). After the 30 min preincubation, cells were co-incubated with endocytosis inhibitors, and either 10^9^ PKH26-labeled sEVs per mL, isolated freshly from HEK293T or SH-SY5Y cells, or equal volumes of medium controls in EBM-2 medium with 1% P/S for 4 h. 1% DPBS and 0.1% DMSO were used as final concentrations in all conditions. Each experiment was repeated three to four times, with three replicates for each condition per experiment.

OGD experiments were performed with modifications as published previously.^9,15^ In brief, 80,000 hCMEC/D3 cells/cm^2^ were seeded onto collagen IV/fibronectin coated 1.0 µm pore-size Greiner 24-well ThinCerts®. Media were changed on days 2 and 5 as described before. In parallel with hCMEC/D3 seeding, 1321N1 astrocytoma cells were seeded into 24-well plates at a density of 15,000 cells/cm^2^ in DMEM++. On day 2 and 5, maintenance medium was changed. On day 6, prior to establishing the co-culture, hCMEC/D3 cells on inserts and 1321N1 cells in wells were washed with DPBS containing Ca^2+^ and Mg^2+^ (#D8662; Sigma-Aldrich) twice, and experimental medium (DMEM with or without glucose (#11966–025; Gibco^TM^) and 1% BSA) was added to each basolateral well and apical insert. For OGD treatment, cells and their blank insert (cell-free) controls in glucose-free medium were placed into a hypoxia chamber (Biospheryx, Parish, NY, USA) with 5% CO_2_, saturated humidity and 0.1% O_2_, for 5 h or 24 h at 37°C. Normoxia controls, including blank inserts without cells, mono-cultured hCMEC/D3 cells and their co-cultures with 1321N1 cells were incubated with DMEM containing glucose in a humified incubator with ambient air O_2_ concentration, 5% CO_2_ at 37°C for the same time.

For OGD model validation studies, mono-cultured BCECs and their co-culture with astrocytoma within the transwell system underwent one of three treatments: (1) 5 h OGD alone, (2) 5 h OGD followed by addition of glucose and 19 h reoxygenation (recovery/normoxia), or (3) 24 h continuous OGD as illustrated (Figure S2A). Each experiment was repeated for three times, with six replicates for each condition per experiment.

In OGD experiments with sEV treatments, on day 6 – prior to the OGD treatment, 10^9^ CTO-labeled sEVs derived from either HEK293T or SH-SY5Y cells or their equivalent medium controls in DPBS were added to the donor compartment – either apically (HEK293T) or basolaterally (SH-SY5Y), in experimental medium supplemented with 1% BSA. The final concentration of DPBS was 1% in all conditions. The sEV-BBB transwell system was exposed to (1) 5 h OGD followed by 19 h recovery, or to (2) 24 h OGD as depicted (Figure S2B). Each experiment was repeated three times, with three replicates for each condition per experiment.

### 2.7 BBB integrity and permeability assessments

Transendothelial electrical resistance (TEER) measurements were performed with an EVOM-3 Voltohmmeter device (World Precision Instruments, Sarasota, FL, USA) and STX2 chopstick electrodes (Merck Millipore). The initial TEER (0 h) was determined after incubating with experimental medium for 40 min at room temperature. TEER was then reassessed after either 5 h or 24 h OGD treatments. For the 5 h OGD samples, glucose was reintroduced, and these samples, along with the normoxia controls, were placed in a normoxia incubator for a 19 h recovery phase. TEER was measured again following this recovery period (at 24 h). TEER values, given as Ω x cm^2^, were calculated by subtracting the mean resistance value (Ω) of three blank ThinCerts® and multiplication with the cell growth surface area (0.336 cm^2^). The paracellular endothelial permeability coefficient (Pe) was measured using fluorescein isothiocyanate (FITC)-dextran 4 kDa (FD4; #FD4; Sigma-Aldrich). The 1 mM stock solution was prepared by dissolving FD4 in PBS, followed by ultrafiltering it with 3 kDa molecular weight cutoff Amicon tubes (#UFC8003; Sigma-Aldrich) to separate from residual, free FITC. The retentate was refilled with PBS to the original volume, sterile-filtered and stored at 4°C with a cover. 10 µM of FD4 (1:100 of the stock solution) was added to the apical medium, and cells were subsequently incubated for 1 h. The collected apical and basolateral media along with stock solutions were pipetted into black 96-well plates (#655097; Greiner Bio-One). Fluorescence was determined using filters (Ex 485/20, Em 535/25), configured with extended dynamic range measurement on a TECAN SPARK 10 M Multimode Microplate Reader (Tecan Group Ltd., Männedorf, Switzerland). The Pe values were calculated using the clearance principle as published before.^9,53^

### 2.8 Flow cytometry and cell viability assay

Flow cytometry of hCMEC/D3 and 1321N1 cells was performed on a CytoFLEX device (Beckman Coulter, Brea, CA, USA) calibrated with CytoFLEX Daily QC Fluorospheres (#B53230; Beckman Coulter) according to the manufacturer’s instructions. Singlet cells were gated using the forward and side scatter signals, and PKH26 or CTO positive cells were determined using the PE channel settings of the instrument (excitation wavelength 488 nm; emission detection using the bandpass filter 585/42BP). In case of treatment with CTO-labeled sEVs, hCMEC/D3 cells treated with media controls served as negative controls for CTO-labeled sEV internalization, and untreated BCECs were stained after the experiment with 10 µM CTO dye for 15 min at room temperature (RT) as positive controls for gating. Flow cytometric data were processed with the Kaluza Analysis software 2.1 (Beckman Coulter). For CTO-labeled sEV uptake assays, the average median fluorescence intensities (MFI) of untreated controls or media controls were subtracted as background from the MFI values of media controls and sEV samples, yielding background-corrected values used for normalization and further calculations. For PKH26-labeled uptake inhibition assays, all samples were normalized to the average MFI of DMSO (vehicle control) and sEV co-treated controls on an experiment-by-experiment basis.

Cell viability was assessed by dye exclusion using a 0.4% Trypan Blue Solution (#15250061; Gibco^TM^). Following the flow cytometric measurements, cells were resuspended before mixing the same volume of cell suspension and Trypan Blue solution. Cells were thoroughly mixed and incubated for 30 s before the first technical measurement and 90 s before the second. The average of both measurements was calculated for each sample. In total, the mean of each condition was determined from *n* = 3 independent experiments, *N* = 9 total samples.

### 2.9 Immunofluorescence staining and confocal microscopy

PKH26-labeled sEVs isolated from SH-SY5Y or HEK293T were added apically to hCMEC/D3 cells on 1.0 µm pore-size inserts. After 24 hours incubation, cells were washed twice with PBS, fixed with 4% paraformaldehyde (PFA; #158127; Sigma-Aldrich) in PBS for 15 min, permeabilised with 0.5% Triton X-100 (#T878; Sigma-Aldrich) in PBS for 5 min and blocked with 0.5% fish skin gelatine (#G7041; Sigma-Aldrich) in PBS for 30 min at RT. The plastic membranes were cut out from the inserts and incubated with primary antibody dilutions overnight at 4°C. The primary antibody against Collagen IV (#AB769, Sigma-Aldrich) was diluted 1:100 in IF block solution. After three DPBS washes, secondary antibody dilution (1:500; #A21447; Life Technologies, Thermo Fisher Scientific) was applied in IF block for 1 h at RT, protected from light. After two PBS washes, 0.1 µg/mL DAPI (4,’6-diamidino-2 phenylindole; #D9542; Sigma-Aldrich) solution in water was applied for 5 min, followed by a final rinse with Milli-Q water. Samples were dried and mounted in EverBrite™ hardset mounting medium (#23003; Biotium Inc., Fremont, CA, USA).

sEV internalization was visualized in hCMEC/D3 cells seeded at a density of 40,000 cells/cm^2^ onto collagen IV and fibronectin-coated coverslips (12 mm, #YX03.2, Carl Roth). Upon reaching 70% confluency, cells were transduced with BacMam reagents following the manufacturer’s instructions: CellLight® Lysosomes-GFP (LAMP1-GFP; #C10596; Thermo Fisher Scientific) or with CellLight® Early Endosomes-RFP (Rab5a-RFP; #C10587; Thermo Fisher Scientific). On the following day, 10^9^/mL PKH26-labeled, SH-SY5Y sEVs or an according media control equivalent were applied, and following 30 min, 4 h- and 24 h-incubations, cells were washed twice with DPBS containing Mg^2+^ and Ca^2+^, fixed with 4% PFA. Samples were not permeabilised. DNA was stained with DAPI, and coverslips were mounted in EverBrite™ onto glass slides as before. Images were captured using a Zeiss LSM 700 (Carl Zeiss AG, Oberkochen, Germany) confocal laser scanning microscope system with a Plan-Apochromat 63x/1.4 Oil DIC objective. Images were processed with the ImageJ/FIJI software.

### 2.10 Gene expression analysis – high-throughput quantitative PCR

Total RNA was isolated from cells lysed in RLT buffer containing 1% β-mercaptoethanol using the “All prep DNA/RNA/protein mini kit” according to the manufacturer’s instructions (#80004; Qiagen). cDNA was generated by reverse-transcription of 250 ng total RNA using the High-Capacity Reverse Transcriptase Kit (#4374967; Applied Biosystems). Selected target genes were preamplified using a Qiagen PCR master mix with the HotStar Plus Taq Polymerase (#203603; Qiagen) as described before.^9,52,54^ The high-throughput qPCR was run with the preamplified cDNAs in a 96 × 96 microfluidic chip setup on a Biomark HD system (Fluidigm®). Threshold cycle (Cq) values were normalized to the geometric mean of the Cq values of four housekeeping genes (ACTB, B2M, GAPDH and PPIA). Relative gene expression levels (x-fold values) were calculated by normalizing the log2-fold changes to normoxia, mono-culture samples. The investigated target- and housekeeping genes and transcriptional changes are listed in supplemental tables S1-S4. Heatmap generation and hierarchical clustering were performed using R software with pheatmap and viridis libraries. In the heatmaps, the value of each target was scaled to the range of −2 to +2 using the “viridis” color scale.

### 2.11 Statistical analysis

Results are presented as mean ± SD, if not indicated otherwise. Statistical outliers were identified using Grubb’s test. Data were analyzed using one-way ANOVA followed by pairwise Holm-Šidak’s multiple comparison post hoc tests, or unpaired t-tests where appropriate. High-throughput qPCR results were analyzed using 2-∆Cq values with the same statistical approach using R software with dplyr, tidyr, and multcomp packages. Statistical tests and graphical visualizations were performed with GraphPad Prism 8.0 (Dotmatics, Boston, MA, USA), except for high-throughput qPCR data which used R software. Differences were considered statistically significant at *p* < 0.05.

## 3. Results

### 3.1 Establishment and characterization of the invitro BBB co-culture OGD model

To investigate the impact of OGD on BBB integrity and paracellular permeability, an *in vitro* BBB co-culture model comprising hCMEC/D3 BCECs cultured in 24-well cell culture inserts on membranes with 1.0 µm pores, and 1321N1 astrocytoma cells seeded in the basolateral wells, was established. Three experimental time points were analyzed: 5 h OGD representing the acute phase of ischemic stroke, 5 h OGD followed by 19 h recovery phase in normoxia with glucose supply, and 24 h OGD to achieve a maximal insult (Figure S2A).

**Figure 1.**
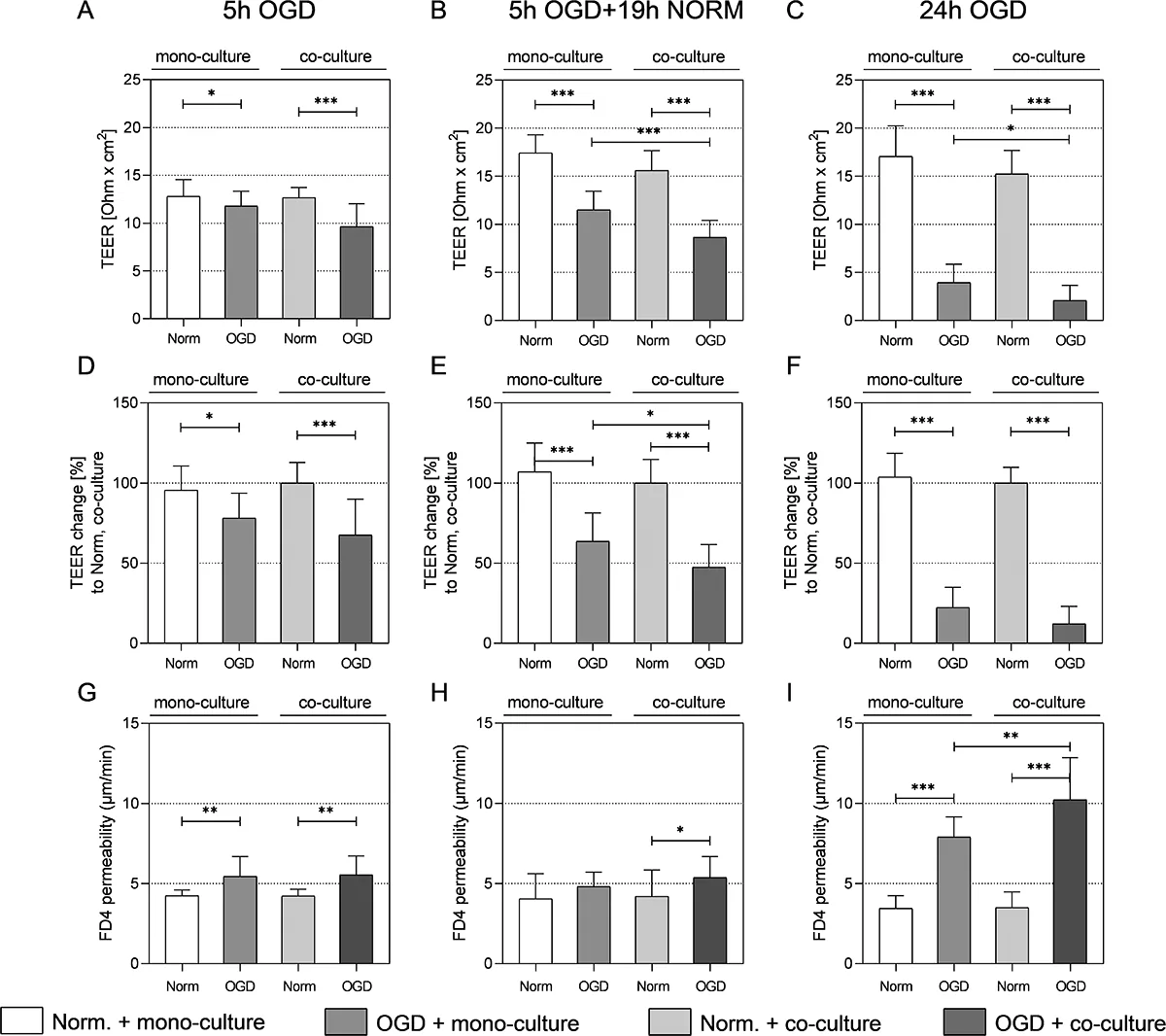
Barrier integrity assessment in an *in vitro* BBB co-culture model under ischemic conditions. (A-F) Transendothelial electrical resistance (TEER) and its changes in mono-cultured hCMEC/D3 cells (1.0 µm inserts) and co-cultures with 1321N1 astrocytoma cells after (A, D) 5 h OGD, (B, E) 5 h OGD followed by 19 h reoxygenation (normoxia), and (C, F) 24 h OGD. TEER values were normalised to normoxia (Norm.) and co-culture conditions, expressed as a percentage. (G-I) Fluorescein isothiocyanate-dextran 4 kDa (FITC-dextran 4 kDa, FD4) permeability measured after (G) 5 h OGD, (H) 5 h OGD followed by 19 h normoxia, and (I) 24 h OGD. 1% BSA was present in both compartments. Data were analyzed using Grubbs’ test and one-way analysis of variance (ANOVA), followed by Holm-Šidak’s post-hoc tests. Data are presented as mean ± SD, *n* = 16–18, *N* = 3. *, *p* < 0.05; **, *p* < 0.01; ***, *p* < 0.001. Norm, normoxia, OGD, oxygen/glucose deprivation.

After 5 h OGD treatment, TEER dropped significantly in both, mono- and co-cultured hCMEC/D3, to 78.05%±15.61% (*p* = 0.0034) and 67.64%±22.28% (*p* < 0.001), respectively, compared to normoxia controls (Figure 1(A,D)). After the additional 19 h recovery phase, the barrier integrity of mono-cultured 5 h OGD-treated hCMEC/D3 stagnated at around 11.5 Ω x cm^2^ (63.62%±17.61% compared to normoxia co-culture controls), co-cultures exhibited a reduction to 47.47%±14.11% (*p* < 0.001, related to the same controls); while the TEER of normoxia controls increased by 4–6 Ω x cm^2^ compared to the first time point after 5 h (Figure 1(B,E)). Extended OGD exposure for 24 h led to severe barrier disruption, with TEER values reduced to ~ 4 Ω x cm^2^ (22.27%±12.65%) in mono-cultures and ~2 Ω x cm^2^ (12.25%±10.83%) in co-cultures compared to 24 h normoxia controls (*p* < 0.001) (Figure 1(C,F)). Overall, these results indicated an adverse effect of the astrocytoma cells on the BCEC barrier – co-cultures with astrocytoma cells led to a more pronounced decrease of TEER after OGD treatments and an impairment of barrier recovery.

In order to assess the paracellular permeability, the apical to basolateral permeation rate of FD4 was determined. After 5 h OGD, consistent with the observed TEER decrease, FD4 permeability was significantly increased in mono-cultured (5.46 ± 1.23 µm/min) and co-cultured BCECs (5.54 ± 1.19 µm/min) as compared to normoxia controls (mono-culture: 4.25 ± 0.36 µm/min; co-culture: 4.23 ± 0.42 µm/min; *p* < 0.01) (Figure 1(G)). After recovery, FD4 permeability remained elevated in OGD-treated cells (mono-culture: 4.81 ± 0.90 µm/min, n.s. *p* > 0.05; co-culture: 5.38 ± 1.31 µm/min, *p* < 0.05) compared to normoxic controls (mono-culture: 4.05 ± 1.57 µm/min; co-culture: 4.21 ± 1.64 µm/min) (Figure 1(H)). Following 24 h OGD, FD4 permeability was highly increased in both mono-cultured (7.90 ± 1.26 µm/min) and co-cultured BCECs (10.23 ± 2.62 µm/min) compared to normoxia controls (mono-culture: 3.45 ± 0.80 µm/min; co-culture: 3.50 ± 0.98 µm/min; *p* < 0.0001) (Figure 1(I)) and co-cultured BCECs exhibited significantly higher permeability than mono-cultured cells (*p* = 0.003). Overall, these results indicated that astrocytoma cells aggravate the effects of OGD on the barrier integrity of co-cultured hCMEC/D3 cells.

### 3.2 Transcriptomic changes in the BBB model in response to OGD

**Figure 2.**
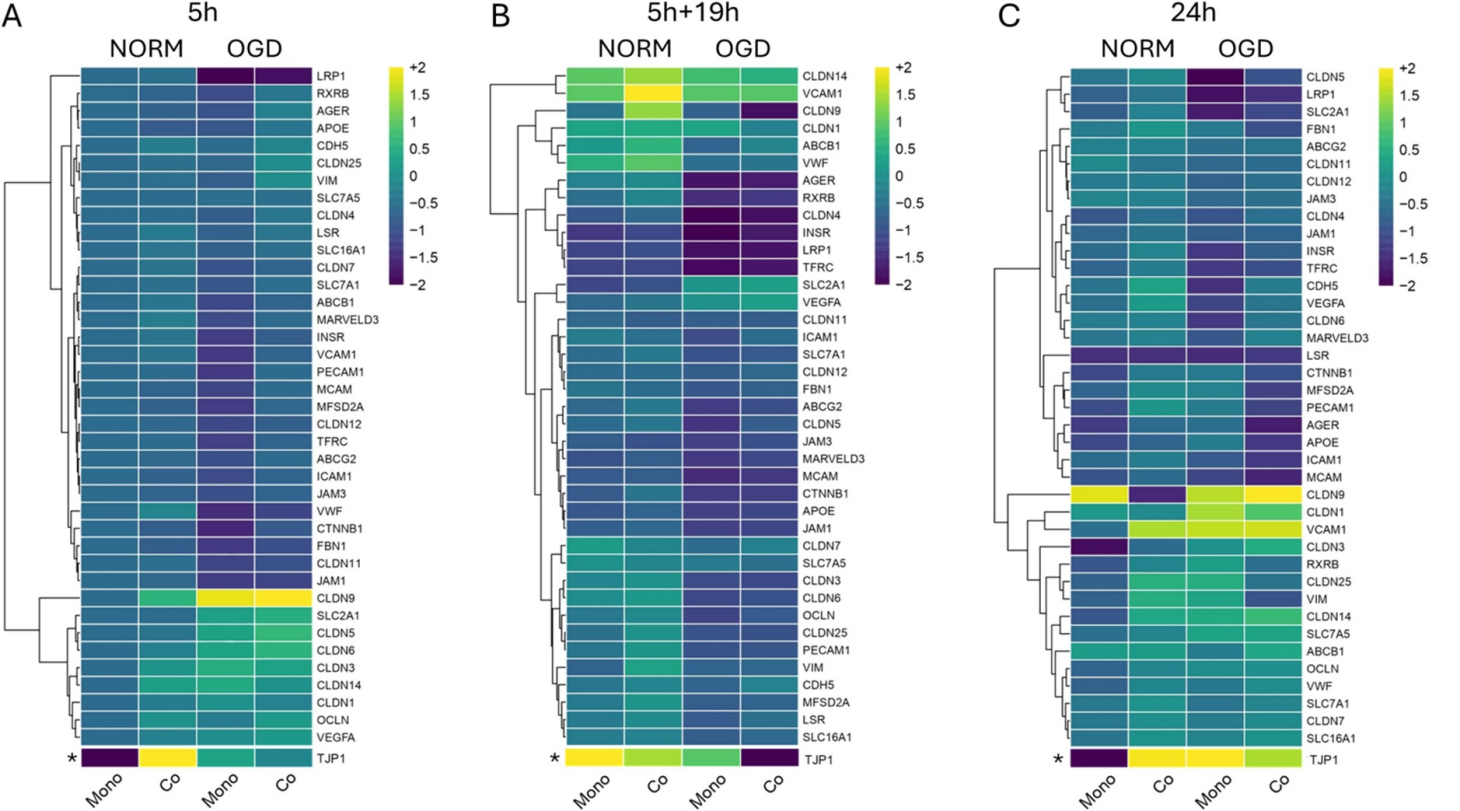
Molecular changes in hCMEC/D3 cells in the 1321N1-hCMEC/D3-based co-culture system under normoxic versus OGD conditions. A comprehensive qPCR chip analysis for BBB-relevant genes encoding tight junction proteins, transporters, endothelial and inflammatory markers. The heatmap and hierarchical clustering of mRNA expression from hCMEC/D3 cells treated with (A) 5 h OGD, (B) 5 h OGD followed by 19 h normoxia, (C) 24 h OGD. 1% BSA was present in both compartments. Threshold cycle (Ct) values were normalised to the geometric mean of 4 housekeeping genes (ACTB, B2M, GAPDH and PPIA) and to mono-culture, normoxia treatment after 5 h OGD. X-fold expression of the mean values of three independent experiments are displayed using the color scale “viridis,” with 2 (yellow) indicating a high expression and −2 (purple) indicating a low expression value. *TJP1 is separately visualized due to high fold-change values that would compress the dynamic range of the heatmap scale. The color code legend is added and formatted in log-scale. Supplementary tables S1, S2 and S4 display the x-fold changes for each condition (Table S1: 5 h OGD; Table S2: 5 h OGD followed by 19 h normoxia; Table S4: 24 h OGD conditions). Data were analyzed using one-way analysis of variance (ANOVA), followed by Holm-Šidak’s post-hoc tests. Heatmap generation and hierarchical clustering were performed using R software with pheatmap and viridis libraries; statistical analysis was conducted using dplyr, tidyr, and multcomp packages. Data are presented as mean, *n* = 5–6, *N* = 3. *, *p* < 0.05; **, *p* < 0.01; ***, *p* < 0.001.

Acute OGD exposure (5 h) induced substantial transcriptional changes with similar patterns in both mono- and co-cultures (Figure 2(A), Table S1). Several tight junction-related proteins showed significant upregulation after OGD treatment, including CLDN5 (mono-cultures: 3.28-fold, *p* = 0.01; co-cultures: 5.53-fold, *p* = 0.0034), CLDN6 (mono-cultures: 3.53-fold, *p* = 0.0038; co-cultures: 5.02-fold, *p* = 0.0006),CLDN9 (mono-cultures: 29.65-fold; co-cultures: 35.67-fold, *p* = 0.0034) and ZO-1 (TJP1) (mono-cultures: 7.68-fold; co-cultures: 13.53-fold), while other claudins and tight junction proteins were downregulated: CLDN7 (mono-cultures: 0.6-fold, *p* = 0.023), CLDN11 (mono-cultures: 0.43-fold; co-cultures: 0.55-fold, *p* < 0.001), CLDN12 (mono-cultures: 0.47-fold, *p* < 0.0001, co-cultures: 0.76-fold, *p* = 0.043), JAM1 (mono-cultures: 0.39-fold, *p* < 0.0001; co-cultures: 0.45-fold, *p* = 0.0011). Additionally, two genes known to be regulated under OGD, SLC2A1 (GLUT1) (mono-cultures: 3.1-fold, *p* = 0.0006; 4.39-fold, co-cultures: *p* < 0.0001) and VEGFA (mono-cultures: 2-fold, co-cultures: 2.75-fold, *p* = 0.0129) were found upregulated, as expected. In mono-cultures, ABC transporters ABCB1 (PGP) (0.43-fold, *p* = 0.028) and ABCG2 (BCRP) (0.54-fold, *p* = 0.002) showed significant downregulation. Control co-cultures did not induce significant transcriptional changes in hCMEC/D3 cells, except for OCLN (normoxia: 2.34-fold, *p* = 0.019; OGD: 1.87-fold; *p* = 0.028) and SLC7A1 (OGD: 0.62-fold; *p* = 0.044), which showed significantly lower gene expression. Concordantly, hierarchical clustering analysis revealed OGD exposure as the primary clustering parameter (Figure 2(A)).

After the recovery phase tight junction proteins including OCLN (mono-culture: 0.7-fold, *p* = 0.021; co-culture: 0.94-fold, *p* = 0.0001), CLDN3 (co-culture: 0.75-fold, *p* = 0.01), CLDN4 (mono-culture: 0.35-fold, *p* < 0.0001; co-culture: 0.41-fold, *p* = 0.0009), CLDN6 (mono-culture: 0.77-fold, *p* = 0.0007; co-culture: 0.84-fold, *p* = 0.0018) and JAM1 (mono-culture: 0.69-fold, *p* = 0.0001; co-culture: 0.73-fold, *p* = 0.0012) were found significantly downregulated in both, mono- and co-culture conditions (Figure 2(B), Table S2). ABCG2 expression remained suppressed (mono-culture: 0.67-fold, *p* < 0.0001; co-culture: 0.82-fold, *p* = 0.0007), whereas SLC2A1 and VEGFA expression stayed at elevated levels in both culture conditions (SLC2A1: mono-culture: 2.11-fold; co-culture: 2.35-fold, *p* < 0.0001; VEGFA: mono-culture: 1.99-fold; co-culture: 2.33-fold, *p* = 0.006).

The analysis of 1321N1 astrocytoma cells following 5 h OGD and 19 h normoxia revealed similar gene expression changes: VEGFA (5.56-fold, *p* = 0.0019) and the transporters SLC2A1 (3.31-fold, *p* = 0.001) and SLC7A5 (LAT1, 1.89-fold, *p* = 0.005) were upregulated, whereas JAM1 (0.58-fold, *p* = 0.017) and CLDN10 transcript variant a (tva) (0.47-fold, *p* = 0.04) were significantly reduced as compared to normoxia controls (Figure S3, Table S3).

After 24 h of OGD exposure, co-cultures, did not exhibit any statistically significant transcriptional changes, and also in mono-cultures, only few transcripts, CLDN5, CLDN6 and CDH5, became significantly downregulated (0.38-fold, *p* = 0.012, 0.6-fold, *p* = 0.017, and 0.56-fold, *p* = 0.046, respectively). Interestingly, SLC2A1 was downregulated in mono-cultures after prolonged OGD (0.48-fold, *p* = 0.0054), contrary to its upregulation after 5 h of OGD exposure and following the combined 5 h OGD and 19 h restoration (Figure 2(C), Table S4).

Altogether, cells reacted to acute (5 hours) OGD by transcriptional changes in a number of BBB related genes, which – interestingly – persisted throughout a 19 h normoxic recovery phase, suggesting that OGD damage can have long-lasting effects on gene expression in the investigated model. The OGD treatment for 24 h may have arrested the cells due to energy deprivation, in particular in the co-culture setup, impairing any gene regulatory activities.

### 3.3 Extracellular vesicle release under OGD conditions

**Figure 3.**
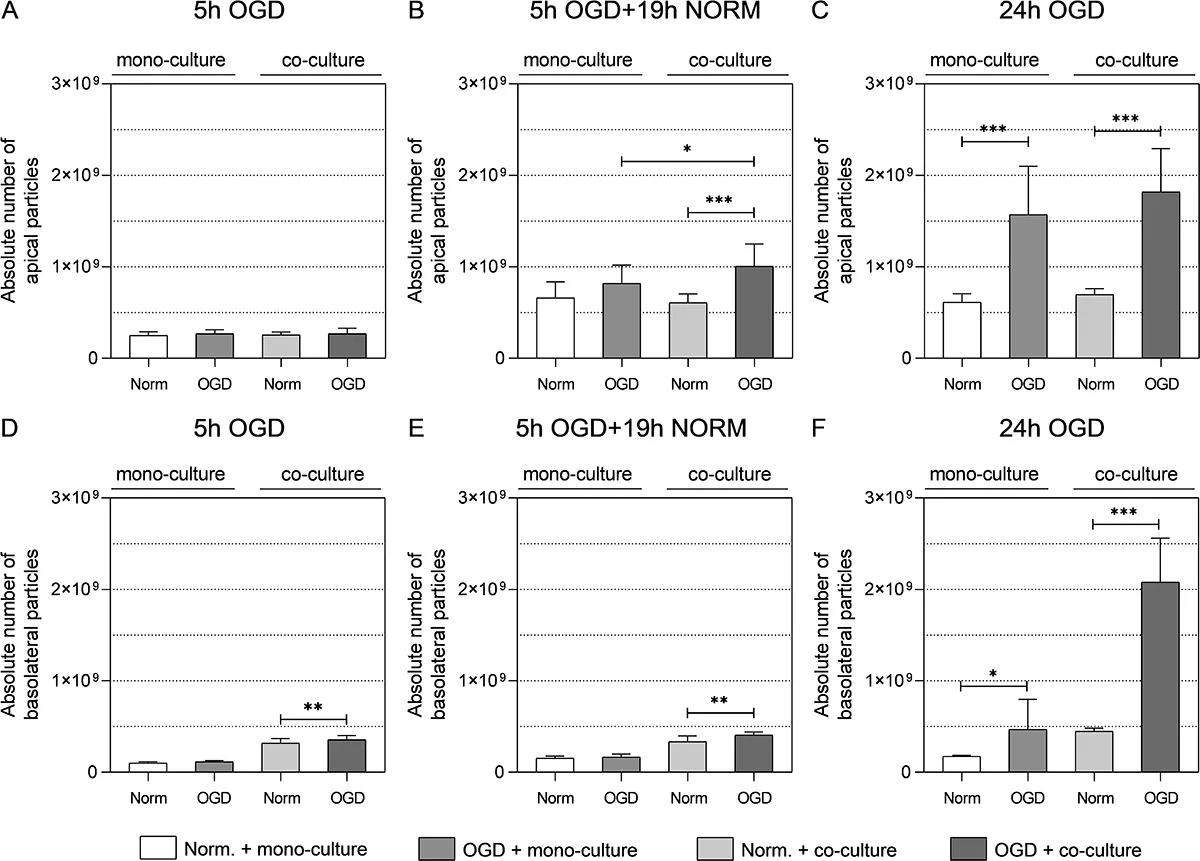
Particle release in the *in vitro* BBB co-culture model under ischemic conditions. The number of particles released (A-C) apically and (D-F) basolaterally was quantified using nanoparticle tracking analysis (NTA) under the following conditions: (A, D) after 5 h OGD, (B, E) after 5 h OGD followed by 19 h reoxygenation/normoxia, and (C, F) after 24 h OGD or normoxia (Norm.). 1% BSA was present in both compartments. Data were analyzed using Grubbs’ test and one-way analysis of variance (ANOVA), followed by Holm-Šidak’s post-hoc tests. Data are presented as mean ± SD, *n* = 18, *N* = 3. *, *p* < 0.05; **, *p* < 0.01; ***, *p* < 0.001. Norm, normoxia, OGD, oxygen/glucose deprivation.

To investigate EV secretion under OGD conditions, particle concentration and size in both, apical and basolateral compartments, were quantified. After 5 h OGD treatment, particle concentration in the apical compartment remained unchanged as compared to normoxia controls (Figure 3(A)) but reduced median particle sizes were observed (normoxia: 175.4–179.3 nm; OGD: 159.0–165.1 nm) (Figure S4A). Following a 19 h recovery in normoxia, apical media of co-cultured cells contained significantly elevated particle concentrations compared to normoxia co-cultures (1.65-fold; *p* < 0.001) (Figure 3(B)), whereas particle concentrations in mono-cultures remained unchanged. Also, particle sizes were similar across all conditions (184.3–187.6 nm) (Figure S4B). Extended OGD exposure (24 h) induced an approximately threefold increase in particle release in mono- and co-cultures as compared to the respective normoxia controls (*p* < 0.001) (Figure 3(C)). Similar to the observations after 5 h OGD, a reduced median particle size was measured (from 190.3 nm to 175.6 nm) (Figure S4C).

Apparently, 1321N1 astrocytoma cells released EVs, hence the basolateral media in co-cultures contained 3–4-fold higher particle concentrations than mono-cultures across all experiments (Figure 3(D-F)). Most likely, the vesicles released by 1321N1 cells are larger than EVs produced by hCMEC/D3 cells, as in co-cultures an increased median particle size was observed (Figure S4D-F). This was also reflected by the median size of purified sEVs: hCMEC/D3 cells released sEVs with a median size of 127.55 nm, whereas 1321N1 sEVs had a median diameter of 145.9 nm (Table S5).

OGD exposure significantly increased particle release on the basolateral side in the co-culture setup, after 5 h OGD (1.12-fold; *p* = 0.006), 5 h OGD +19 h recovery (1.22-fold; *p* = 0.0001) and 24 h OGD (4.67-fold; *p* < 0.0001) (Figure 3(D-E)), whereas in mono-cultured BCECs only after 24 h OGD higher numbers of particles on the basolateral side were found (2.68-fold; *p* = 0.004) (Figure 3(F)). These findings demonstrate a progressive increase in vesicle secretion and particle release (likely small debris of apoptotic/necrotic cells contribute to the total numbers) during extended OGD exposure.

### 3.4 Comparison of uptake and permeation of a panel of cancer sEVs across the BBB model

Small EVs from eight human cancer cell lines originating from various organs (brain, breast, lung, skin; Figure S1), were purified using a combination of ultrafiltration and size exclusion chromatography and characterized subsequently. All isolates displayed typical sEV characteristics: (1) presence of sEV markers on protein level (CD9, CD81, Alix, TSG101; Figure S5B), and the absence of cellular contaminants confirming isolates purity (GM130, β-actin; Figure S5B), (2) size distribution ranging from 129.94 ± 8.28 nm to 151.39 ± 13.08 nm, and (3) negative zeta potentials (−42.34 ± 9.27 mV to −19.72 ± 5.12 mV) (Figure S5C-D).^55^ A representative sample of a HEK293 sEV preparation was processed for cryo-transmission electron microscopy (cryo-TEM), visualizing the characteristic double membrane of the isolated vesicles, as a validation of the applied isolation methodology (Figure S5A).

In order to reveal potential differences in uptake (and/or permeation) efficiency of sEVs originating from different cell types, a series of experiments was performed, testing their internalization by hCMEC/D3 cells from both, the apical (equivalent to the blood compartment in the physiological setting) and basolateral (brain parenchyma) side. The uptake of CellTracker Orange (CTO)-labeled sEVs was quantified by flow cytometry and calculated as the difference between the median fluorescence intensity of treated cells and the respective media controls. It is important to note, that media controls (described in detail in the material and methods section) allowed to estimate the unspecific background caused by particle isolation and fluorescent dye labeling procedures (Figure S6, untreated vs. media controls), which was crucial to exclude false-positive interpretation of sEV uptake and permeation measurements.

**Figure 4.**
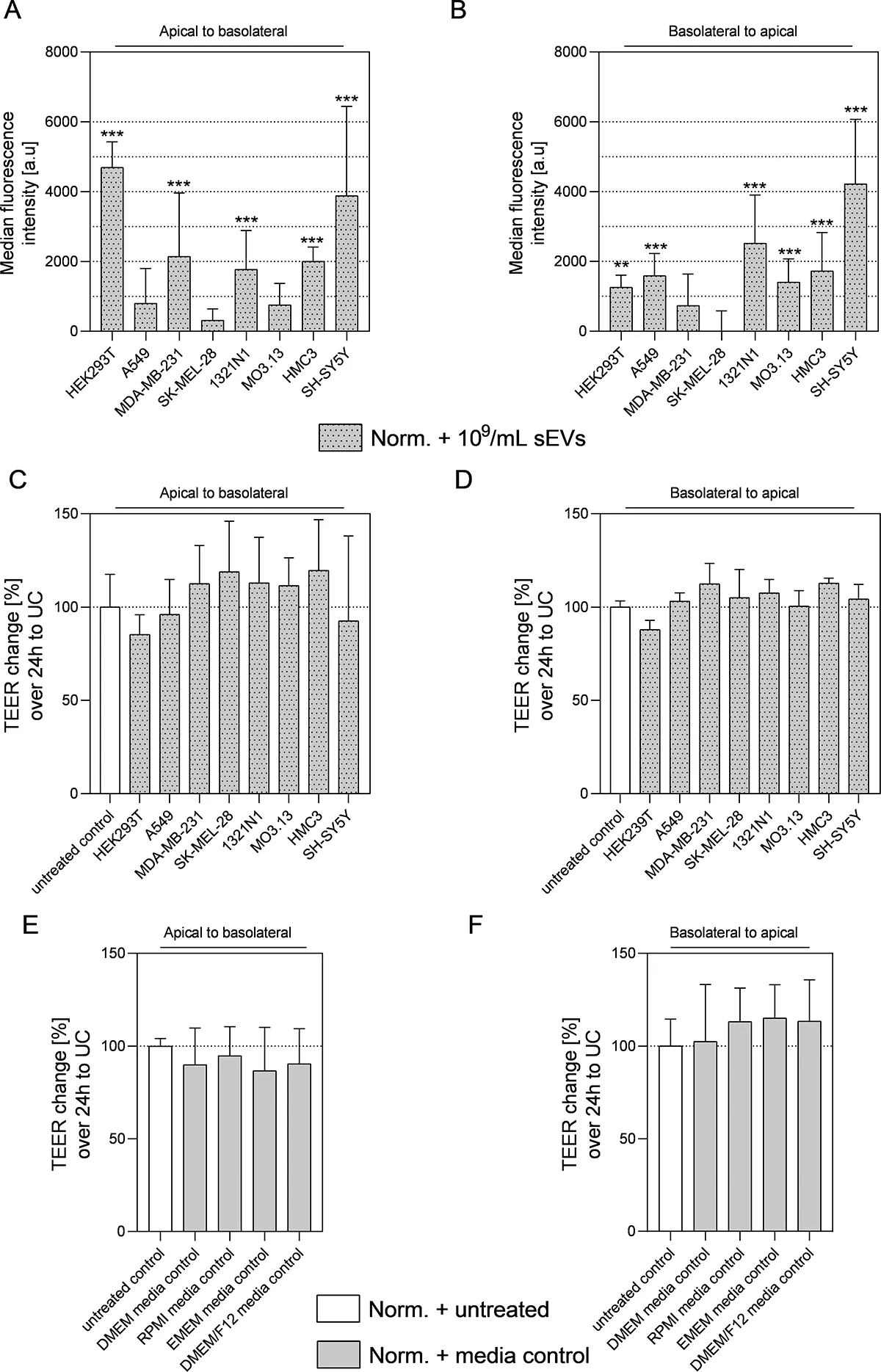
The uptake of human sEVs by BCECs depends on the sEV origin and donor compartment, while barrier integrity of BCECs is unaffected. (A-B) uptake of CellTracker Orange (CTO)-labeled sEVs (10^9^/mL) from eight human cell lines, applied (A) apically or (B) basolaterally to 1.0 µm pore-size inserts over 24 h. Data are shown as median fluorescence intensity corrected for each corresponding media control background. (C-D) transendothelial electrical resistance (TEER) changes relative to the untreated control (UC) following (C, E) apical or (D, F) basolateral application of (C-D) CTO-labeled sEVs (10^9^/mL) or (E-F) each corresponding media control particles with 1% BSA in the receiver compartment over 24 h. Media controls: DMEM (HEK293T, A549, MDA-MB-231, 1321N1, and MO3.13), RPMI medium (SK-MEL-28), EMEM (HMC3), and DMEM/F12 (SH-SY5Y). Data were analyzed using Grubbs’ test and one-way analysis of variance (ANOVA), followed by Holm-Šidak’s post-hoc tests. Data are presented as mean ± SD, *n* = 6–18, *N* = 3. *, *p* < 0.05; **, *p* < 0.01; ***, *p* < 0.001.

When applied apically, five sEV types were significantly internalized by BCECs as compared to their media controls (Figure 4(A)), in descending order of fluorescence intensity [a.u.]: HEK293T (4697 ± 731; *p* < 0.0001), SH-SY5Y (3882 ± 2556; *p* < 0.0001), MDA-MB-231 (2151 ± 1808; *p* < 0.0001), HMC3 (2003 ± 412; *p* = 0.0007), and 1321N1 (1774 ± 1112; *p* = 0.0003). With basolateral application (Figure 4(B)), the order of uptake efficiency changed: SH-SY5Y (4219 ± 1854; *p* < 0.0001), 1321N1 (2571 ± 1380; *p* < 0.0001), HCM3 (1726 ± 1097; *p* = 0.0006), A549 (1589 ± 639; *p* = 0.0006), MO3.13 (1411 ± 663; *p* = 0.0006) and HEK293T (1257 ± 351; *p* = 0.0014). The uptake of SK-MEL-28 sEVs was not significant above media controls from either direction.

To corroborate that the applied sEVs were internalized via specific uptake pathways hCMEC/D3 cells were pre-treated with endocytosis inhibitors before incubation with labeled sEVs. In particular nystatin, filipin III and cytochalasin D significantly reduced the uptake of both, HEK293T and SH-SY5Y-derived sEVs, pointing to caveolae-mediated endocytosis and macropinocytosis as primary pathways (Figure S7).

In addition, barrier integrity (TEER) of the cell layer and sEV permeation across the model were measured. None of the tested sEV samples affected the TEER significantly at the concentration applied (10^9^/mL) (Figure 4(C-F)) and while sEVs readily permeated through empty inserts (up to 18.46%±5.63% after 24 h) (Figure S8A-B), no permeated sEVs could be detected on the opposite side of the BBB model using nanoparticle tracking analysis (Figure S8C-D), indicating that (transcellular) permeation of vesicles within 24 h under normoxic conditions was very low.

**Figure 5.**
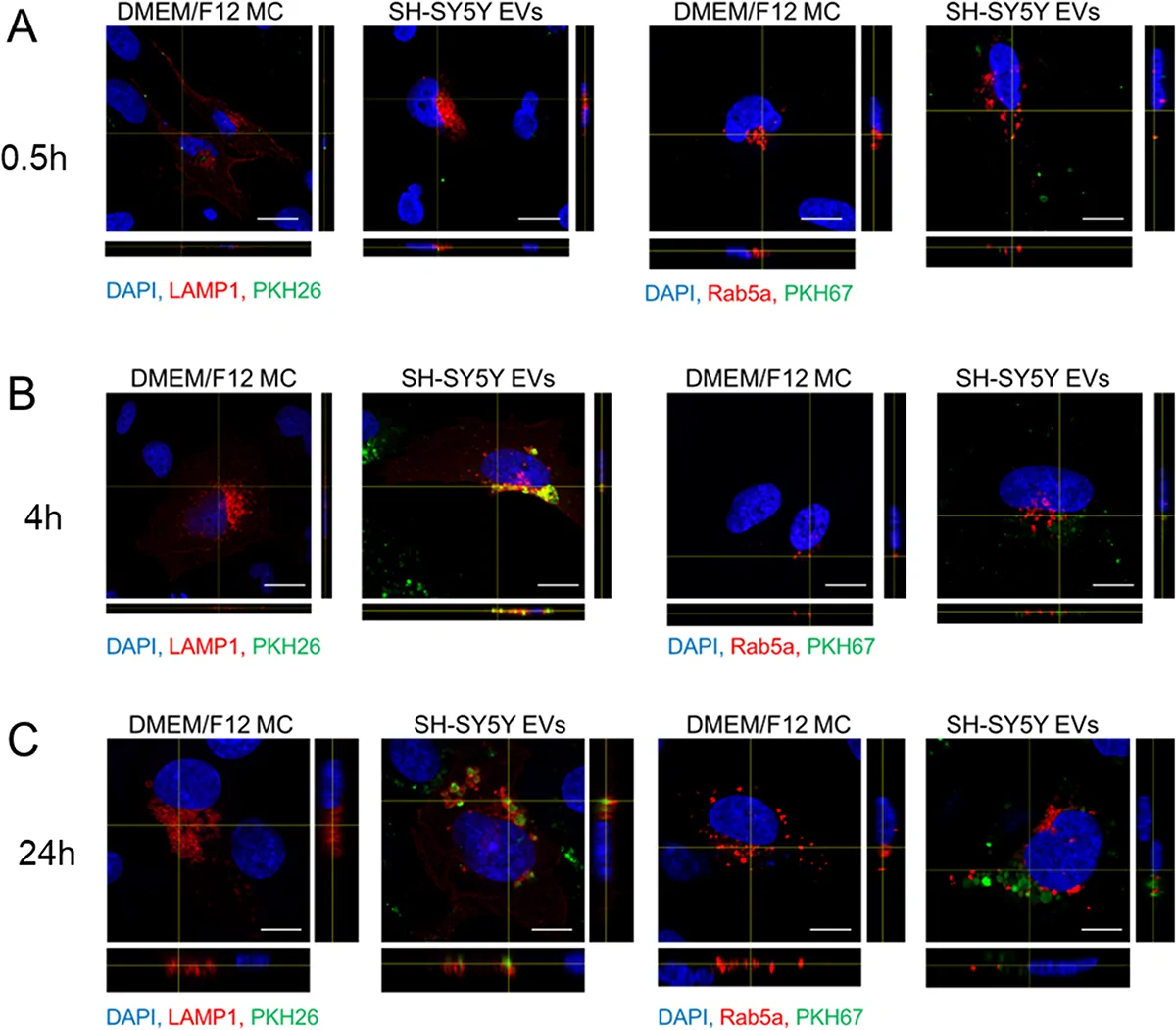
SH-SY5Y sEVs end up in the lysosomal structures at the BBB. (A-C) Representative orthogonal images (XY in the middle, XZ below, YZ on the left) of the presence or absence of colocalisation of LAMP1 (lysosomal) and Rab5a (early endosomal) markers with PKH26-labeled SH-SY5Y sEVs after 0.5 h, 4 h and 24 h. (A) After 30 minutes, a few sEV spots are visible, showing colocalisation sites neither with LAMP1 nor Rab5a. (B) After 4 h sEV-treatment, sEVs are internalised and colocalised with LAMP1 signal. (C) After 24 h incubation, sEVs are in and around the lysosomes. DAPI was used for staining nuclei. Scale bar: 10 µm.

Using confocal microscopy, a progressive accumulation of SH-SY5Y-derived sEVs in lysosomes could be detected (colocalisation increased after 4 h till 24 h incubation) (Figure 5). Furthermore, z-stacks confirmed that both HEK293T and SH-SY5Y sEVs were internalized by BCECs but remained confined to the cells and the extracellular matrix with no evidence of transcellular transport (Figure S9).

Hence, these results show, that the applied sEVs were efficiently taken up by BCECs, but were degraded in the lysosomal compartment, rather than being transported and released at the opponent side of the cell layer, at quantities which could be detected reliably by nanoparticle tracking analysis.

### 3.5 Uptake but no permeation of HEK293T and SH-SY5Y sEVs were detectable under OGD conditions

Previous experiments have shown that sEVs (1) are internalized by BCECs with different efficiency depending on their cellular origin and application direction. HEK293T sEVs showed the highest uptake from the apical side, while SH-SY5Y were efficiently internalized from both directions, and (2) no significant number of the applied sEVs permeated across the intact BBB cell layer under normoxic conditions. In order to test whether sEV uptake and/or transport might be changed in case of a compromised barrier integrity, sEVs of HEK293T and SH-SY5Y cells were applied to the BBB model under OGD conditions.

**Figure 6.**
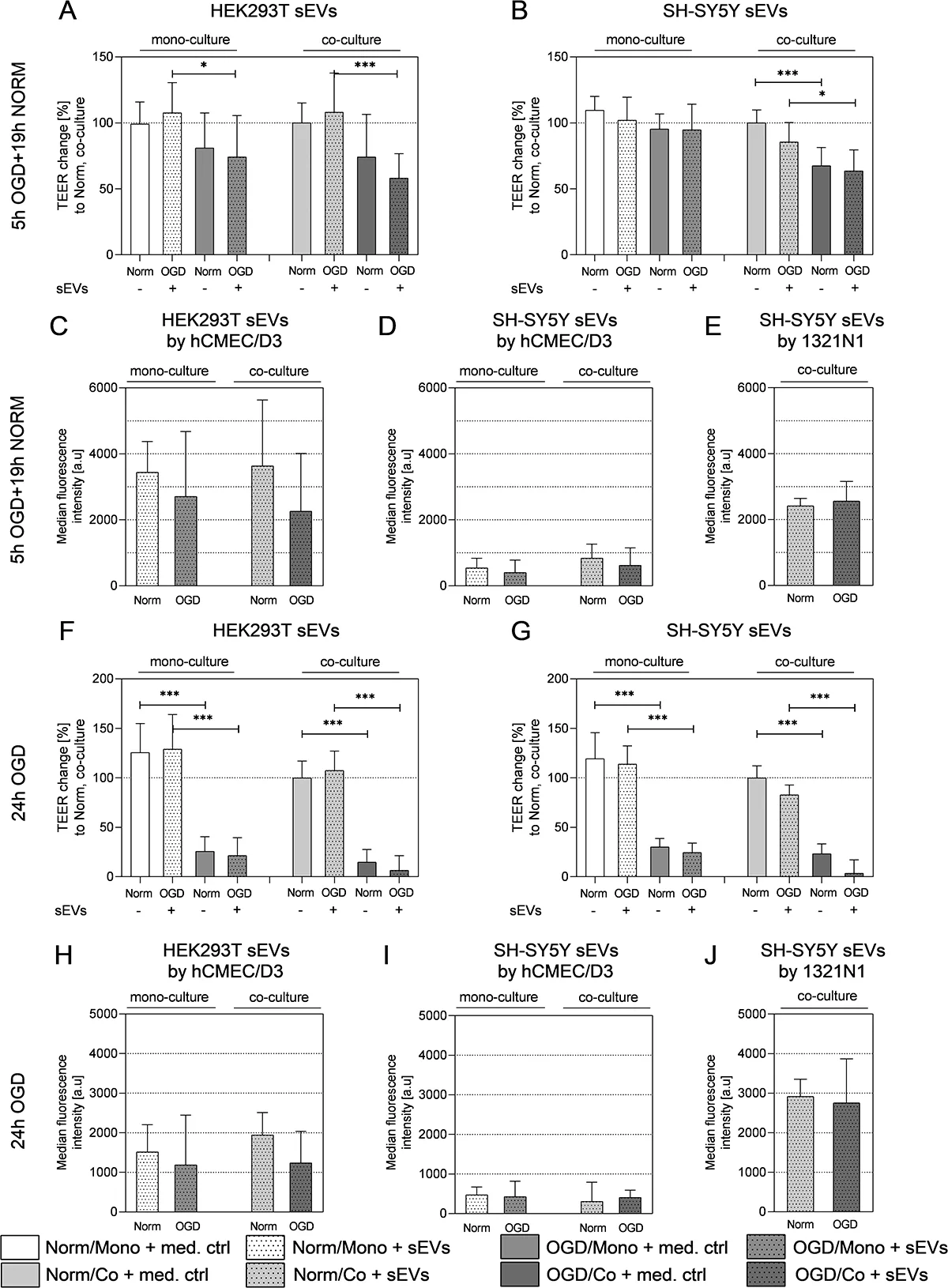
OGD duration determines barrier integrity loss, but does not influence sEV uptake patterns. (A-B, F-G) TEER changes in mono-cultured hCMEC/D3 cells and co-cultures with 1321N1 astrocytoma cells with exogenous HEK293T or SH-SY5Y sEVs under (A-B) 5 h OGD followed by 19 h normoxia or (F-G) 24 h OGD. Values were normalised to normoxia and co-culture (%). (C-E, H-J) Uptake of CellTracker Orange (CTO)-labeled sEVs (10^9^/mL) applied apically (HEK293T-derived) or basolaterally (SH-SY5Y-derived) to hCMEC/D3 cells or 1321N1 astrocytoma cells after (C-E) 5 h OGD followed by 19 h normoxia or (H-J) 24 h OGD versus normoxia. Data are shown as median fluorescence intensity corrected for each corresponding media control background. 1% BSA was present in both compartments. Data were analysed using Grubb’s test and one-way ANOVA, followed by Holm-Šidak’s post-hoc tests, except (e, j) with applied unpaired t-test. Data are presented as mean ± SD, *n* = 8–9, *N* = 3. *, *p* < 0.05; **, *p* < 0.01; ***, *p* < 0.001. Norm, normoxia, OGD, oxygen/glucose deprivation, mono, mono-culture, co, co-culture, med. ctrl, medium control, sEVs, 10^9^ sEVs/mL.

Similar to previous experiments, a moderate TEER decrease was observed after 5 h OGD and following a 19 h recovery phase in normoxia, in mono- and co-culture conditions, as compared to normoxia controls (Figure 6(A-B)). Treatment with HEK293T- or SH-SY5Y-derived sEVs exerted no statistically significant effect on TEER changes. Uptake of HEK293T sEVs by the BCECs was slightly, but not significantly, reduced under OGD conditions (Figure 6(C)), a trend which was also observed for basolaterally applied SH-SY5Y sEVs (Figure 6(D)). The level of sEV uptake by co-cultured 1321N1 cells was similar in normoxia and after OGD treatment (Figure 6(E)). Furthermore, no permeation of applied sEVs was detectable, in either direction (Figure S10A-B).

In addition, uptake and permeation of sEVs were tested on a severely disrupted BCEC barrier, after 24 h of OGD exposure. Similar to previous experiments, extended OGD severely disrupted barrier integrity across all conditions (Figure 6F-G), as TEER values decreased to 3.44%±13.57% to 24.56%±9.55% compared to normoxic conditions (*p* < 0.0001). Barrier disruption was accompanied by a drop in cell viability (19.67%±4.33% to 46.67%±7.0%) compared to normoxia controls (89.56%±3.05% to 93.11%±3.44%) (Figure S11). Despite these drastic effects on cellular functions, neither uptake of sEVs was significantly changed between conditions, nor was permeation of labeled sEVs detected (Figure 6H-J, Figure S10C-D).

In summary, although readily internalized by BCECs, sEVs of HEK293T and SH-SY5Y did not affect barrier integrity (as measured by TEER) under normoxia and OGD conditions, at the applied concentrations. The uptake efficiency of sEVs did not significantly change in any condition, neither did applied sEVs permeate – even not across severely disrupted BBB – in quantities which could be reliably detected by NTA.

### 3.6 Particle release in the BBB co-culture model upon co-treatment with OGD and sEVs

**Figure 7.**
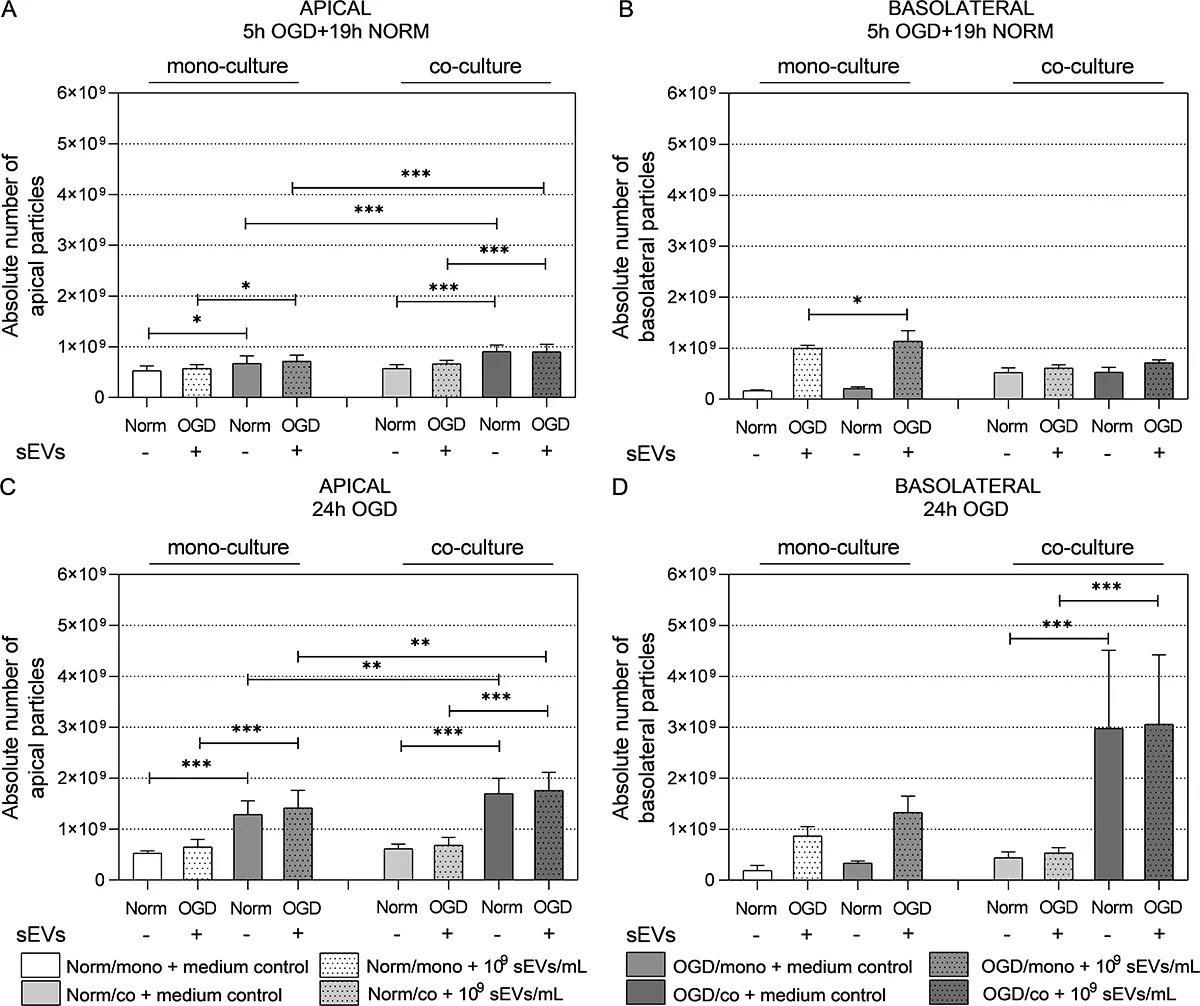
Besides the enhanced particle release by OGD, basolateral SH-SY5Y sEV treatment demonstrates astrocytic internalization. Following treatment with 10^9^ sEVs/mL of SH-SY5Y sEVs, or their respective medium control particles, the number of apically (A, C) and basolaterally (B, D) released particles was quantified using nanoparticle tracking analysis (NTA) under the following conditions: apical and basolateral release after (A-B) 5 h OGD followed by 19 h normoxia (Norm.) with sEVs and (C-D) 24 h OGD with sEVs, respectively. 1% BSA was present in both compartments. Data were analyzed using Grubbs’ test and one-way analysis of variance (ANOVA), followed by Holm-Šidak’s post-hoc tests. Data are presented as mean ± SD, *n* = 9, *N* = 3. *, *p* < 0.05; **, *p* < 0.01; ***, *p* < 0.001. Norm, normoxia, OGD, oxygen/glucose deprivation, mono, mono-culture, co, co-culture.

OGD treatment significantly increased particle release into both, apical and basolateral compartments (Figure 7, Figure S12). Following 5 h OGD and 19 h recovery, basolateral SH-SY5Y treatment revealed significant differences in particle secretion between mono- and co-cultured hCMEC/D3 cells (*p* < 0.0001) (Figure 7(A)), indicating that the co-cultured astrocytoma cells modulated endothelial vesicle release. No differences were observed in cases of additional basolateral treatment with SH-SY5Y sEVs (Figure 7(A,B)). When SH-SY5Y sEVs were added to basolateral co-cultures, recovery of sEVs was reduced as compared to mono-cultures under both normoxic and OGD conditions (Figure 7(B)), likely due to internalization of SH-SY5Y sEVs by 1321N1 cells. Similarly, after 24 h OGD, basolateral co-culturing significantly impacted particle release into the apical compartment (increased up to 2.76-fold; *p* < 0.0001) regardless of additional sEV treatment (Figure 7(C)). With co-cultured astrocytoma cells, the BCECs exhibited a strongly increased vesicle/debris release (showing up to 6.74-fold increase; *p* < 0.0001) (Figure 7(D)). A second experimental setup applying combined treatments with OGD and HEK293T-derived sEVs yielded similar results: consistently, OGD treatment significantly increased particle release by BCECs (up to 2.62-fold after 5 h OGD plus 19 h recovery and up to 12.20-fold after 24 h OGD), whereas the presence of HEK293T-sEVs exerted no obvious effects (Figure S12).

In summary, OGD increased particle release. Co-culture with astrocytoma cells enhanced endothelial particle release, but no sEV permeation across the hCMEC/D3 layers was detected even after 24 h OGD treatment.

## 4. Discussion

In this study, the effects of OGD and the role of sEVs at the BBB were investigated in an *in vitro* co-culture model comprising the human brain endothelial cell line hCMEC/D3 (representing BCECs) and 1321N1 astrocytoma cells. BBB-related gene expression, sEV release, and the uptake and permeability of sEVs derived from eight different cell types were examined under normoxic and ischemic stroke conditions. Specifically, OGD effects on sEV release from the endothelial cells and astrocytes, sEV uptake by these cell types, and permeation across the BBB model were assessed at distinct time points during cerebral ischemia *in vitro*.

hCMEC/D3 cells have been extensively used in various culture setups for modeling the BBB *in vitro*. Barberio et al.^37^ co-cultured hCMEC/D3 cells with 1321N1 astrocytoma cells to build a highly usable and robust platform for studying the NVU in a transwell setup. In OGD models, hCMEC/D3 cells have been previously employed both in mono-cultures^34,56^ and in co-culture setups^9^ with rat-derived C6 glioma cells and primary human astrocytes and pericytes.^9,14,15^ In comparison to those co-culture models, 1321N1 cells have the advantage of their human origin (avoiding species differences in the system) and their higher stability over passages and thus better reproducibility compared to primary cells.

In order to facilitate the study of sEVs, in particular the potential permeation across a BBB model, a previously established, optimized transwell model for such approaches, employing inserts with larger pores (1.0 µm instead of regularly used 0.4 µm pore sizes) and an adapted media composition with 1% BSA for enhanced sEV recovery, was used.^22^ Hence, it should be taken into account, that barrier-forming cells, such as the used hCMEC/D3, may exhibit lower TEER values when grown on 1.0 µm- as compared to 0.4 µm pores as it was shown earlier.^57^ Besides the pore size differences, the presence of astrocytes was shown to be necessary to model the pathophysiologically relevant barrier disruption within a 4-6 h time window.^9^ As demonstrated before, the co-culture with astrocytoma cells led to a stronger OGD effect (e.g. reduced TEER values) compared to OGD treated mono-cultures. Additionally, given that 1321N1 cells are tumor-derived, secreting more VEGF^58^ and other growth factors than primary astrocytes, they induce stronger barrier breakdown suitable for studying BBB interactions with sEVs under even more compromised states.

Interestingly, transcriptomic analysis revealed upregulation of several tight junction proteins (CLDN5, CLDN6, CLDN9, ZO-1) measured after the acute OGD phase, particularly in mono-cultures, which could be interpreted as an early compensatory mechanism to maintain barrier integrity during initial ischemic stress, whereas in other model setups tight junction components were found downregulated after OGD treatments,^9,59,60^ indicating that some effects can be model dependent. Consistent with previous reports, glucose transporter (GLUT1, SLC2A1) and VEGFA were upregulated, representing adaptive responses to nutrient deprivation and hypoxia.^9,59,60^

Similar as observed before, hCMEC/D3 failed to recover barrier functionality after OGD and a recovery phase in normoxia with glucose supply.^56^ Different results were reported for mono-cultured human-induced pluripotent stem cell-derived BCECs, which fully restored barrier integrity after 16 h of OGD treatment followed by a 12 h recovery phase with glucose and oxygen supplementation.^61^ Regarding molecular changes in our model, samples after the 19 h recovery phase revealed significant downregulation of multiple genes encoding tight junction proteins including OCLN, CLDN3, CLDN4, CLND6 and JAM1, consistent with the observed persistent barrier dysfunction. Meanwhile, both GLUT1 and VEGFA expression remained significantly elevated, suggesting ongoing metabolic adaptation and angiogenic stimulus despite glucose and oxygen availability. Altogether these data suggest that 5 h OGD in the 1321N1-hCMEC/D3 co-culture model may trigger transcriptomic alterations lasting at least for 24 h, which could prevent complete recovery of barrier integrity.

Extended 24 h OGD resulted in complete barrier breakdown and cell death. Importantly, results of this study indicate that astrocytoma cells do not significantly influence BCEC viability during OGD, suggesting that the enhanced barrier disruption observed in co-cultures could be mediated through secreted factors.^62–64^ Transcriptomic analysis showed downregulation of genes encoding junctional proteins (CLDN5, CLDN6, and CDH5) during prolonged OGD, corresponding to barrier breakdown.

Altogether, this study highlights the response of neurovascular cell types to microenvironmental changes induced by OGD, including barrier integrity, regulation of gene expression and release of extracellular vesicles. OGD increased the release of vesicles from both BCECs (up to threefold) and astrocytes (up to fivefold), which aligns with clinical observations of elevated circulating EV levels 24 h post-ischemic stroke^29^ and increased sEV release from cytokine-activated BCECs.^30^ Following ischemic insults, endothelial cells undergo significant inflammatory stress, which promotes endothelial EV release carrying typical markers correlated with stroke, such as ICAM-1.^40^ In the current study, it was observed that the cells released vesicles with reduced size under OGD conditions, consistent with previous reports in hypoxic cancer cells.^39^ Overall, both, increased vesicle release and altered EV cargo seem to be cellular stress responses to OGD.

Interrupted oxygen and glucose supply under ischemic stroke leads to reduced ATP production, which can affect sEV uptake, as an active, energy-dependent process.^19,20,24^ Indeed, sEV uptake by NVU cells was changed in an OGD-dependent manner: BCECs showed reduced uptake capacity for HEK293T-derived sEVs in both, short-term and prolonged OGD protocols, whereas 1321N1 astrocytoma cells reacted with an enhanced uptake during 5 h OGD + 19 h recovery but reduced uptake during 24 h of OGD, potentially due to the restored metabolic activity after a recovery phase.^56^ An important conclusion from the performed uptake studies was the basic necessity to include EV labeling controls in such experimental setups. When comparing the uptake efficiency of eight different cell line derived sEVs, SK-MEL-28 consistently ranked lowest regarding uptake (Table S6-S8, Figure 4(A-B)), but at the same time, exhibited inefficient fluorescent labeling – which infers that the apparent low internalization might partly reflect insufficient detection of fluorescently labeled vesicles in the cells, rather than lower cellular uptake. Furthermore, median fluorescence intensity (MFI) values of untreated- and media controls revealed substantially different background, dependent on the respective basal medium (DMEM, DMEM/F12, EMEM, RPMI; Figure S6). These findings highlight the critical importance of using stringent control conditions when using (lipid-dye based) fluorescently labeled sEVs to prevent artifacts caused by divergent background and unspecific labeling.

Several parameters influence the uptake of sEVs, including EV surface molecules and protein corona,^65,66^ vesicle size and concentration, interaction time, and type and physiological status of the recipient cell.^67–69^ Jurgielewicz et al.^70^ conducted a comprehensive study on EV uptake kinetics and specificity using imaging flow cytometry, where it was found that EV uptake is not only an active process, but also dose and time dependent. To measure quantifiable amount of internalized HEK293T EVs by recipient HEK293T cells, minimum 4 h of incubation was necessary, while the EV uptake signal peaked between 4 and 12 h which was corresponding to the optimized incubation times for uptake measurements in this study.^70^

At the BBB, four different uptake pathways for sEVs have been identified: macropinocytosis,^19,21,28^ adsorptive-mediated endocytosis,^71^ caveolae-mediated endocytosis^19,28,72^ and clathrin-mediated endocytosis.^19,21^ In the current study, inhibition of the macropinocytosis (5 µM cytochalasin D) and caveolae-mediated endocytosis pathways (25 µM nystatin; 1.5 µM filipin III) significantly reduced the number of internalized HEK293T or SH-SY5Y sEVs after 4 h of incubation (applied at a concentration of 10^9^ sEVs/mL) (Figure S9).

Following internalization, EVs could be recycled to the plasma membrane, subjected to lysosomal degradation, or transported through the cell (transcytosis).^73^ In the presented model, transcytosis (i.e. permeation of labeled sEVs across the endothelial layer) could not be detected under any of the tested conditions, even after severe barrier disruption following 24 h OGD. Confocal microscopy revealed no evidence of transcellular transport of applied sEVs across the hCMEC/D3 cell layer, but a progressive accumulation of labeled sEVs near lysosomes was observed. These results align with observations, suggesting that lysosomal degradation represents the predominant fate of sEVs at the BBB, even under pathological conditions.^74^ While this *in vitro* model indicated that lysosomal degradation is the predominant fate of sEVs at the BBB, it remains possible that additional signals from the brain parenchyma *in vivo* could influence EV trafficking. These additional cues might promote abluminal release of EVs rather than degradation, a possibility that cannot be fully captured in the current transwell setup.

In order to exclude that the used transwell system poses a physical obstacle which prevents sEV permeation, an additional experiment was conducted using mannitol treatment on mono-cultured BCECs. Mannitol, a hyperosmotic agent clinically used to reduce cerebral edema, opens the BBB by inducing osmotic stress and tight junction disassembly.^75^ Applying 1.4 M mannitol resulted in nearly complete cell death (0.68% viability) and abolishment of TEER (Figure S13A-B). Under this condition, 2.03% of HEK293T-derived sEVs permeated across the disrupted cell layer, as compared to 13.72% in cell-free inserts and 0% in untreated controls after 24 h (Figure S13C). This experiment confirmed, that it is technically possible to detect sEV permeation across a damaged cell barrier at numbers which can be reliably quantified by NTA. Furthermore, cells even when dead, or extracellular matrix produced by the cells, seemed still to impair EV permeation when compared to cell-free inserts.

Permeation of sEVs across biological barriers remains a critical question, with significant methodological challenges *in vitro*. While it was recently demonstrated for the first time using a particle-based detection method (nanoscale flow cytometry) that EVs purified from blood can traverse endothelial barriers,^76^ several technical parameters should be considered for the interpretation of these data and future studies using fluorescently labeled EVs: according to preliminary data up to ~74% of signal in media control samples was caused by unspecific background, when stopping the PKH26 labeling with 1% BSA in PBS. Concordantly, it was demonstrated before, that quenching with 10% BSA or 20% EV-depleted FBS produced high levels of false positive dye particles, and control samples became indistinguishable from PKH26-stained sEVs.^77^

Secondly, media containing FBS or BSA can affect the properties of BBB cells, e.g. BSA was shown to increase and stabilize TEER in hCMEC/D3 cells.^22^ Therefore, labeling procedures, experimental media (especially supplements and inherent particle- and fluorescent background) and stringent control samples need to be considered to minimize the risk for technical artifacts. The methodological complexity of such experimental setups might account for the heterogeneity of results reported on the permeation of EVs across the BBB.

## 5. Limitations and concluding remarks

The vesicle measurements in this study relied primarily on NTA, and fluorescently labeled sEVs (CTO and PKH26), which have inherent limitations, i.a. variable labeling efficiencies and formation of nonspecific aggregates. On the other hand, NTA allows to detect single, intact vesicles in contrast to most other methods, which employ the measurement of (labeled) EV cargo. Furthermore, in this study, sEVs were applied at concentrations which are physiologically relevant for single cell type-derived vesicles in the bloodstream and cerebrospinal fluid (10^9^ particles per mL) – which could be insufficient for detecting rare permeation events.

In conclusion, this study provides a comprehensive characterization of a human BBB co-culture model, revealing the response of brain endothelial- and astrocytoma cells under ischemic conditions by changes in gene expression and particle release.

## Supplementary Material

Supplemental Material
