# Supplementary material for: Permeation of small extracellular vesicles across a human blood-brain barrier transwell model remains below particle detection limits, even under oxygen/glucose deprived conditions
Source: Tissue Barriers. 2026 Jan 9;14(3):2610016. doi: 10.1080/21688370.2025.2610016 (PMC13540092; doi:10.1080/21688370.2025.2610016)
Supplement: Supplementary file 1 [file KTIB_A_2610016_SM4595.docx]

Figure S1: **Human cell line panels used for sEV studies.** Eight cell lines grouped into two panels: 1) Blood-circulating panel: HEK293T (human embryonic kidney cells) as the control cell line, and three brain-metastasising tumour cell lines: A549 (lung adenocarcinoma), SK-MEL-28 (skin melanoma), and MDA-MB-231 (breast adenocarcinoma). 2) Brain cell panel: MO3.13 (oligodendrocytes), HMC3 (microglia), 1321N1 (astrocytoma), and SH-SY5Y (neuroblastoma). The figure was created using Biorender.com. Following cell lines were kindly provided by Roland Foisner, MFPL, Vienna, Austria (MDA-MB-231, SK-MEL-28, HEK293T), David Male, UK (MO13.3). Sigurd Krieger, AKH, Vienna, Austria (A549) and Hannes Steinkellner, MUW, Vienna, Austria (SH-SY5Y).

Figure S2: **Establishing, validating and co-treating a human *in vitro* BBB co-culture model under ischemic conditions with sEVs.** (A) Three treatment conditions were tested in both mono-culture (hCMEC/D3 cells) and co-culture (hCMEC/D3 and 1321N1 astrocytoma cells): (1) 5h oxygen-glucose deprivation (OGD), (2) 5h OGD followed by 19h recovery/normoxia, or (3) 24h OGD. Barrier integrity was assessed by measuring transendothelial electrical resistance (TEER) at 0h, 5h, and 24h, as well as with fluorescein isothiocyanate-dextran 4 kDa (FITC-dextran 4, FD4) permeability. Additional endpoint measurements included scatter-mode nanoparticle tracking analysis (S-NTA) for sEV-like particle release and cell lysis for molecular studies. (B) Two treatment conditions were tested in the presence of sEVs: mono- and co-cultures underwent either (4) 5h OGD followed by 19h recovery/normoxia or (5) 24h OGD. CellTracker Orange (CTO)-labelled HEK293T sEVs (10⁹/mL) were applied apically, and CTO-labelled SH-SY5Y sEVs (10⁹/mL) were applied basolaterally, with corresponding medium controls. Barrier integrity was monitored by TEER at 0h, 5h, and 24h. Endpoint measurements included barrier integrity (TEER), sEV uptake (flow cytometry), permeation and particle release (fluorescence- and scatter-mode NTA). The figure was created using Biorender.com.

Figure S3: **Molecular changes in 1321N1 cells under normoxic versus OGD conditions.** A comprehensive qPCR chip analysis for BBB-relevant genes encoding tight junction proteins, transporters, endothelial and inflammatory markers. The heatmap and hierarchical clustering of mRNA expression from 1321N1 cells treated with 5h OGD followed by 19h normoxia. 1% BSA was present in both compartments. Threshold cycle (Ct) values were normalised to the geometric mean of 4 housekeeping genes (ACTB, B2M, GAPDH and PPIA) and to mono-culture, normoxia treatment. X-fold expression of the mean values of three independent experiments are displayed using the colour scale “viridis”, with 2 (yellow) indicating a high expression and -2 (purple) indicating a low expression value. The colour code legend is added and formatted in log-scale. Supplementary table S3 displays the x-fold changes in mRNA expression in 1321N1 cells after 5h OGD followed by 19h normoxia. Data were analysed using one-way analysis of variance (ANOVA), followed by Holm-Šidak’s post-hoc tests. Heatmap generation and hierarchical clustering were performed using R software with pheatmap and viridis libraries; statistical analysis was conducted using dplyr, tidyr, and multcomp packages. Data are presented as mean, n=3, N=3. *, p<0.05; **, p<0.01; ***, p<0.001.

Figure S4: **Median size of released particles in the *in vitro* BBB co-culture model under ischemic conditions.** The median size of particles released (A-C) apically and (D-F) basolaterally was quantified using nanoparticle tracking analysis (NTA) under the following conditions: (A, D) after 5h OGD, (B, E) after 5h OGD followed by 19h reoxygenation/normoxia, and (C, F) after 24h OGD or normoxia (Norm). 1% BSA was present in both compartments. Data were analysed using Grubbs' test and one-way analysis of variance (ANOVA), followed by Holm-Šidak’s post-hoc tests. Data are presented as mean ± SD, n=18, N=3. *, p<0.05; **, p<0.01; ***, p<0.001. Norm, normoxia, OGD, oxygen/glucose deprivation.

Figure S5: **Characterisation of sEVs derived from eight human cell lines**. (A) Representative image of a single HEK293T double membrane bound vesicle observed by cryo-transmission electron microscopy (cryo-TEM) (as described in Špilak et al., 2024). Scale bar = 100 nm. (B) Immunoblots showing sEV-enriched markers (Alix, TSG101, CD81, CD9) and negative markers (GM130, β-actin). 15 µg pf protein was loaded per sample. (C-D) Size distribution and surface charge characteristics of sEVs: mean of the (C) median diameter (nm) and (D) zeta potential (mV) measurements. Data are presented as mean ± SD, n=4-9, N=2-3. HEK293T (human embryonic kidney cells), A549 (lung adenocarcinoma), SK-MEL-28 (skin melanoma), MDA-MB-231 (breast adenocarcinoma), MO3.13 (oligodendrocytes), HMC3 (microglia), 1321N1 (astrocytoma), and SH-SY5Y (neuroblastoma) cells.

Figure S6: **Media control correction is essential for accurate assessment of human sEV uptake by BCECs**. (A-B) Uptake of CellTracker Orange (CTO)-labelled sEVs (10^9^/mL) from eight human cell lines and their corresponding media controls, applied (A) apically or (B) basolaterally to 1.0 µm pore-size inserts over 24h. Data are shown as the difference in median fluorescence intensity between untreated control correction and media control background correction. Media controls: DMEM (HEK293T, A549, MDA-MB-231, 1321N1, and MO3.13), RPMI medium (SK-MEL-28), EMEM (HMC3), and DMEM/F12 (SH-SY5Y). Data are presented as mean ± SD, n=6-18, N=3. *, p<0.05; **, p<0.01; ***, p<0.001. Norm, normoxia.

Figure S7: **Effects of endocytosis inhibitors on sEV internalisation**. hCMEC/D3 cells were pretreated with the following endocytosis inhibitors at the selected concentrations for 30 min: DMSO (vehicle control), chlorpromazine (CPZ, 28 µM), nystatin (NYS, 25 µM), filipin III (FIL, 1.5 µM), cytochalasin D (CCD, 5 µM), chloroquine (CLQ, 30 µM), followed by 4h incubation of 10^9^/mL PKH26-labelled (A) SH-SY5Y or (B) HEK293T sEVs. Cellular uptake was measured by flow cytometry and normalised to DMSO (vehicle control) and sEV co-treatment. Data were analysed using Grubbs' test and one-way analysis of variance (ANOVA), followed by Holm-Šidak’s post-hoc tests. Data are presented as mean ± SD, n=6-12, N=3-4. *, p<0.05; **, p<0.01; ***, p<0.001.

Figure S8: **Permeation and median size of human sEVs through optimised blank and cell-seeded transwell models**. (A) Permeation and (B) median size of CellTracker Orange (CTO)-labelled sEVs (10^9^/mL) from eight cell lines, applied apically (AP to BL) or basolaterally (BL to AP) to blank, cell-free 1.0 µm pore-size inserts were quantified using fluorescence nanoparticle tracking analysis (F-NTA) over 24h. (C-D) CTO-labelled sEVs were measured in the stock solutions and receiver samples ((C) basolateral and (D) apical) in cell-seeded transwell system using F-NTA after 24h. Data were normalised to the stock solution. 1% BSA was added to the receiver compartment. Outliers were identified using Grubbs' test. Data are presented as mean ± SD, n=6-15, N=3. n.d.: number of CTO+ sEVs below the detection limit (>5 particles/frame). AP to BL: apical-to-basolateral, BL to AP: basolateral-to-apical direction.

Figure S9: **No crossing of sEVs through this BBB model *in vitro*.** (A-D) Representative orthogonal images (XY in the middle, XZ below, YZ on the left) with 3-4 selected spots of the localisation of an extracellular matrix protein, collagen IV (Coll IV) and PKH26-labelled (A-B) HEK293T and (C-D) SH-SY5Y sEVs after 24h. (B, D) sEVs are present around the nuclei (counterstained with DAPI) and on the level of the extracellular matrix (Coll IV), but no permeating sEV signal is observed. Scale bar: 10 µm (XY) and 5 µm (XZ and YZ).

Figure S10: **Even after barrier breakdown, no detectable amount of sEVs derived from HEK293T and SH-SY5Y cells can permeate through BCECs on 1.0 µm pore-size inserts under OGD conditions**. Fluorescently labelled sEVs were measured in the stock solutions, donor and receiver compartments using F-NTA after (A-B) 5h OGD followed by 19h normoxia (Norm) or after (C-D) 24h OGD. Data were normalised to the stock solutions (ST). 1% BSA was added to both compartments. Data were analysed using Grubbs' test and one-way analysis of variance (ANOVA), followed by Holm-Šidak’s post-hoc tests. Data are presented as mean ± SD, n=6-9, N=3. *, p<0.05. n.d.: number of CTO+ sEVs below the detection limit (>5 particles/frame). AP to BL: apical-to-basolateral, BL to AP: basolateral-to-apical direction. Norm, normoxia, OGD, oxygen/glucose deprivation.

Figure S11: **24-hour OGD-treatment reduces cell viability regardless of sEV co-treatment.** Viability of (A-B) hCMEC/D3 cells in mono- and co-culture with (C-D) 1321N1 astrocytoma cells following a 24-hour OGD exposure with (A, C) apically applied HEK293T-derived or (B, D) basolaterally administered SH-SY5Y-derived CTO-labelled sEVs (10^9^/mL) using 1.0 µm pore-size inserts. 1% BSA was present in both compartments. Data were analysed using Grubbs' test and one-way analysis of variance (ANOVA), followed by Holm-Šidak’s post-hoc tests. Data are presented as mean ± SD, n=9, N=3. *, p<0.05; **, p<0.01; ***, p<0.001. Norm, normoxia, OGD, oxygen/glucose deprivation, mono, mono-culture, co, co-culture.

Figure S12: **OGD-driven particle release is the primary source of particles despite of the apically applied HEK293T sEVs.** Following treatment with 10⁹ sEVs/mL of HEK293T sEVs, or their respective medium control particles, the number of apically (A, C) and basolaterally (B, D) released particles was quantified using nanoparticle tracking analysis (NTA) under the following conditions: apical and basolateral release after (A-B) 5h OGD followed by 19h normoxia (Norm) with sEVs and (C-D) 24h OGD with sEVs, respectively. 1% BSA was present in both compartments. Data were analysed using Grubbs' test and one-way analysis of variance (ANOVA), followed by Holm-Šidak’s post-hoc tests. Data are presented as mean ± SD, n=9, N=3. *, p<0.05; **, p<0.01; ***, p<0.001. Norm, normoxia, OGD, oxygen/glucose deprivation, mono, mono-culture, co, co-culture.

Figure S13: **D-mannitol treatment compromises barrier integrity and enables sEV transbarrier permeation while reducing cellular viability.** Following treatment with vehicle control, or 1.4 M D-mannitol in combination with apical application of media control or 10^9^ sEVs/mL HEK293T-derived sEVs, barrier function and sEV permeation were assessed in hCMEC/D3 mono-cultures under the following conditions: (A) cell viability assessed by MTT assay expressed as percentage of vehicle and media control (UT+MC) co-treated samples, (B) transendothelial electrical resistance (TEER) measurements normalised to vehicle and media control (UT+MC) co-treated samples, expressed as percentage, and (C) permeation of fluorescently-labelled HEK293T-derived sEVs across the barrier quantified by F-NTA in the receiver compartment after 24-hour exposure. 1% BSA was present in both compartments. Data were analysed using Grubbs' test and one-way analysis of variance (ANOVA), followed by Holm-Šidak's post-hoc tests. Data are presented as mean ± SD, n=3. ***, p<0.001. MC: media control; UT: untreated – vehicle treated.

**Supplementary Materials and Methods:**

Figure S13-related descriptions:

1. *In vitro* model and mannitol treatment

After establishing the hCMEC/D3 barrier model detailed above, a hyperosmolar mannitol solution (2.8 M stock solution) was prepared in EBM-2 medium containing 1% BSA. Following pH adjustment to 7.4 and sterile filtration using a 0.22 µm filter, 1.4 M mannitol solution was prepared. The cells were treated with this concentration of mannitol at 37°C, 5% CO₂ for 24 hours.

2. BBB integrity assessment

Barrier integrity of mannitol-treated cells was assessed by measuring transendothelial electrical resistance (TEER) at the start of 24-hour incubation (0h) and at the end of the treatment (24h). TEER measurements were performed as described in the main section 2.7.

3. Metabolic activity assay

Following the mannitol experiment, MTT assay was performed to assess the metabolic activity (cell viability) of hCMEC/D3. Cells were treated with 15 µL of 12 mM MTT stock solution (50 mg solid MTT dissolved in 10 mL DPBS; #M2128, Thermo Fisher Scientific) and were incubated for 60 minutes at 37°C. Subsequently cells were washed once ice-cold DPBS, which was completely removed, and sterile filtered DMSO was added to dissolve the formed formazan crystals. To ensure complete dissolution, the plate was incubated for 10 minutes at 37°C. From each insert, twice 100 µL aliquots were transferred to a 96-well plate. Absorbance was measured at 570 nm using an EnSpire plate reader (Perkin Elmer, Shelton, CT, US). Cell viability was calculated as a percentage of the untreated control with media control (UT+MC) values. Untreated cells served as negative controls (100% viability).

References:

Špilak, A., Brachner, A., Friedl, H. P., Klepe, A., Nöhammer, C., & Neuhaus, W. (2024). Effects of small extracellular vesicles derived from normoxia- and hypoxia-treated prostate cancer cells on the submandibular salivary gland epithelium in vitro. *Tissue Barriers*, *13*(1), 1–19. https://doi.org/10.1080/21688370.2024.2347062

Table S1: X-fold changes in the gene expression of housekeeping and target markers from hCMEC/D3 cells after 5h OGD using high-throughput qPCR barrier chip. Threshold cycle (Ct) values were normalised to the geometric mean of the Cq values of four housekeeping genes (ACTB, B2M, GAPDH and PPIA). Relative gene expression levels (x-fold values) were calculated by normalising the log2-fold changes to normoxia, mono-culture samples. Data were analysed separately for each single target using one-way analysis of variance (ANOVA), followed by Holm-Šidak’s post-hoc tests. Data are presented as mean ± SD, n=6, N=3. CLDN8, and CLDN10: n.d. all in all samples or weak expression. Statistical significance: between normoxia and OGD from the same culture type (mono/co-culture): *, p<0.05; **, p<0.01; ***, p<0.001, ****, p<0.0001; and between mono- and co-culture within the same treatment (normoxia/OGD): #, p<0.05; ##, p<0.01; ###, p<0.001; ####, p<0.0001.

| Genes\Treatments | Co-culture hCMEC/D3, Normoxia 5h | Co-culture hCMEC/D3, OGD 5h | Mono-culture hCMEC/D3, Normoxia 5h | Mono-culture hCMEC/D3,  OGD 5h |
| --- | --- | --- | --- | --- |
| ABCB1 | 1,21 ± 0,61 | 0,83 ± 0,27 | 1 ± 0,19 * | 0,48 ± 0,28 |
| ABCG2 | 0,9 ± 0,43 | 0,95 ± 0,34 | 1 ± 0,1 ** | 0,54 ± 0,18 |
| ACTB | 1,54 ± 0,89 | 0,72 ± 0,1 | 1 ± 0,18 | 0,88 ± 0,34 |
| AGER | 0,77 ± 0,63 | 1,54 ± 1,06 | 1 ± 0,21 | 0,61 ± 0,3 |
| APOE | 0,67 ± 0,47 | 1,23 ± 0,84 | 1 ± 0,21 | 0,61 ± 0,32 |
| B2M | 0,88 ± 0,29 | 1,08 ± 0,25 | 1 ± 0,05 | 0,84 ± 0,13 |
| CDH5 | 1,47 ± 0,46 | 1,72 ± 0,25 | 1 ± 0,13 | 0,98 ± 0,14 ### |
| CLDN1 | 1,47 ± 0,99 | 1,64 ± 0,9 | 1 ± 0,44 | 2,35 ± 1,22 |
| CLDN3 | 2,61 ± 2,35 | 3,29 ± 3,04 | 1 ± 0,21 *** | 4,39 ± 1,3 |
| CLDN4 | 0,99 ± 0,47 | 1,23 ± 0,43 | 1 ± 0,06 | 0,72 ± 0,26 |
| CLDN5 | 1,19 ± 0,43 ** | 5,53 ± 1,85 | 1 ± 0,19 * | 3,28 ± 1,39 |
| CLDN6 | 1,56 ± 0,66 *** | 5,02 ± 1,22 | 1 ± 0,2 ** | 3,53 ± 1,33 |
| CLDN7 | 1,14 ± 0,57 | 0,85 ± 0,24 | 1 ± 0,21 * | 0,6 ± 0,14 |
| *CLDN8** | 0 ± 0 | 0,8 ± 0,5 | 0,5 ± 0,7 | 0,36 ± 0,2 |
| CLDN9 | 4,86 ± 5,81 | 35,67 ± 41,99 | 1 ± 0,43 ** | 29,65 ± 13,46 |
| *CLDN10** | 1,04 ± 0 | 5,4 ± 4,16 | 1 ± 0,53 | 1,74 ± 1,34 |
| CLDN11 | 1,03 ± 0,53 | 0,55 ± 0,07 | 1 ± 0,14 **** | 0,43 ± 0,07 |
| CLDN12 | 0,9 ± 0,26 | 0,76 ± 0,19 | 1 ± 0,12 *** | 0,47 ± 0,11 # |
| CLDN14 | 3,18 ± 3,03 | 2,47 ± 2,75 | 1 ± 0,23 ** | 3,92 ± 1,4 |
| CLDN25 | 1,13 ± 0,75 | 1,99 ± 1,28 | 1 ± 0,29 | 0,86 ± 0,36 |
| CTNNB1 | 0,72 ± 0,51 | 0,65 ± 0,52 | 1 ± 0,25 *** | 0,26 ± 0,11 |
| FBN1 | 0,73 ± 0,51 | 0,53 ± 0,28 | 1 ± 0,24 ** | 0,37 ± 0,2 |
| GAPDH | 1,11 ± 0,86 | 1,07 ± 0,15 | 1 ± 0,14 | 0,94 ± 0,25 |
| ICAM1 | 0,78 ± 0,56 | 0,82 ± 0,5 | 1 ± 0,16 * | 0,49 ± 0,28 |
| INSR | 1,2 ± 0,46 | 0,72 ± 0,23 | 1 ± 0,13 **** | 0,39 ± 0,09 # |
| JAM1 | 0,9 ± 0,2 ** | 0,45 ± 0,05 | 1 ± 0,06 **** | 0,39 ± 0,05 |
| JAM3 | 0,86 ± 0,45 | 0,82 ± 0,18 | 1 ± 0,14 *** | 0,53 ± 0,06 # |
| LRP1 | 1,1 ± 0,35 *** | 0,18 ± 0,03 | 1 ± 0,08 **** | 0,13 ± 0,03 |
| LSR | 1,36 ± 0,36 | 1,26 ± 0,48 | 1 ± 0,16 | 0,8 ± 0,43 |
| MARVELD3 | 1,43 ± 1,02 | 0,93 ± 0,32 | 1 ± 0,08 *** | 0,51 ± 0,13 |
| MCAM | 0,82 ± 0,57 | 0,93 ± 0,44 | 1 ± 0,19 ** | 0,43 ± 0,19 |
| MFSD2A | 0,79 ± 0,71 | 0,87 ± 0,53 | 1 ± 0,26 ** | 0,38 ± 0,17 |
| OCLN | 2,34 ± 0,87 | 2,74 ± 0,68 | 1 ± 0,12 # | 1,46 ± 0,62 # |
| PECAM1 | 0,98 ± 0,92 | 0,94 ± 1 | 1 ± 0,25 ** | 0,36 ± 0,24 |
| PPIA | 1,97 ± 1,68 | 1,35 ± 0,89 | 1 ± 0,2 | 2,04 ± 0,9 |
| RXRB | 0,83 ± 0,57 | 1,21 ± 0,6 | 1 ± 0,23 * | 0,51 ± 0,21 |
| SLC2A1 | 0,98 ± 0,55 **** | 4,39 ± 0,8 | 1 ± 0,1 *** | 3,1 ± 0,87 # |
| SLC7A1 | 1,09 ± 0,51 | 1,03 ± 0,26 | 1 ± 0,1 ** | 0,62 ± 0,14 # |
| SLC7A5 | 0,98 ± 0,49 | 1,12 ± 0,27 | 1 ± 0,07 | 1,02 ± 0,35 |
| SLC16A1 | 1,21 ± 0,6 | 1,08 ± 0,23 | 1 ± 0,1 | 0,77 ± 0,21 |
| TFRC | 0,93 ± 0,63 | 0,81 ± 0,21 | 1 ± 0,1 **** | 0,41 ± 0,06 ## |
| TJP1 | 83,12 ± 106,1 | 7,68 ± 13,55 | 1 ± 0,25 | 13,53 ± 20,78 |
| VCAM1 | 1,19 ± 0,74 | 0,85 ± 1,07 | 1 ± 0,35 * | 0,35 ± 0,26 |
| VEGFA | 1,54 ± 0,59 * | 2,75 ± 0,48 | 1 ± 0,21 * | 2 ± 0,57 |
| VIM | 1,11 ± 0,98 | 2 ± 1,59 | 1 ± 0,28 | 0,75 ± 0,4 |
| VWF | 1,75 ± 0,82 * | 0,44 ± 0,18 | 1 ± 0,15 **** | 0,28 ± 0,12 |

Table S2: X-fold changes in the gene expression of housekeeping and target markers from hCMEC/D3 cells after 5h OGD followed by 19h normoxia conditions using high-throughput qPCR barrier chip. Threshold cycle (Ct) values were normalised to the geometric mean of the Cq values of four housekeeping genes (ACTB, B2M, GAPDH and PPIA). Relative gene expression levels (x-fold values) were calculated by normalising the log2-fold changes to normoxia, mono-culture samples. Data were analysed separately for each single target using one-way analysis of variance (ANOVA), followed by Holm-Šidak’s post-hoc tests. Data are presented as mean ± SD, n=6, N=3. CLDN8, and CLDN10: n.d. all in all samples or weak expression. Statistical significance: between normoxia and OGD from the same culture type (mono/co-culture): *, p<0.05; **, p<0.01; ***, p<0.001, ****, p<0.0001; and between mono- and co-culture within the same treatment (normoxia/OGD): #, p<0.05; ##, p<0.01; ###, p<0.001; ####, p<0.0001.

| Genes\Treatments | Co-culture hCMEC/D3, Normoxia 5+19h | Co-culture hCMEC/D3, OGD 5+19h | Mono-culture hCMEC/D3, Normoxia 5+19h | Mono-culture hCMEC/D3,  OGD 5+19h |
| --- | --- | --- | --- | --- |
| ABCB1 | 3,23 ± 1,82 | 1,66 ± 0,75 | 2,17 ± 0,96 | 1,08 ± 0,25 |
| ABCG2 | 1,41 ± 0,22 *** | 0,82 ± 0,11 | 1,12 ± 0,13 *** # | 0,67 ± 0,05 # |
| ACTB | 1,25 ± 0,65 | 0,99 ± 0,49 | 0,82 ± 0,09 | 0,98 ± 0,32 |
| AGER | 1,77 ± 1,13 | 0,46 ± 0,18 | 1,65 ± 0,82 * | 0,43 ± 0,29 |
| APOE | 1,07 ± 0,61 | 0,75 ± 0,43 | 0,89 ± 0,46 | 0,69 ± 0,51 |
| B2M | 0,98 ± 0,17 | 0,97 ± 0,17 | 1,07 ± 0,18 | 0,89 ± 0,12 |
| CDH5 | 1,69 ± 0,58 | 1,6 ± 0,51 | 1,36 ± 0,41 | 1,08 ± 0,29 |
| CLDN1 | 2,77 ± 1,16 | 1,56 ± 0,4 | 2,57 ± 0,94 | 2,58 ± 0,97 |
| CLDN3 | 1,96 ± 0,62 * | 0,75 ± 0,3 | 1,73 ± 0,96 | 0,8 ± 0,42 |
| CLDN4 | 1,1 ± 0,28 *** | 0,41 ± 0,12 | 0,91 ± 0,1 **** | 0,35 ± 0,12 |
| CLDN5 | 1,37 ± 0,53 | 0,97 ± 0,32 | 1,18 ± 0,5 | 0,59 ± 0,17 |
| CLDN6 | 2,2 ± 0,6 ** | 0,84 ± 0,27 | 1,85 ± 0,31 *** | 0,77 ± 0,29 |
| CLDN7 | 1,72 ± 0,7 | 1,57 ± 0,35 | 2,31 ± 0,53 ** | 1,18 ± 0,3 |
| *CLDN8** | 0 ± 0 | 0 ± 0 | 0 ± 0 | 0 ± 0 |
| CLDN9 | 6,04 ± 9,74 | 0,42 ± 0,47 | 1,3 ± 2,17 | 1,04 ± 1,49 |
| *CLDN10** | 0 ± 0 | 0 ± 0 | 0 ± 0 | 0 ± 0 |
| CLDN11 | 0,96 ± 0,23 | 0,98 ± 0,2 | 1,11 ± 0,27 | 1,01 ± 0,21 |
| CLDN12 | 1,21 ± 0,24 | 1,11 ± 0,3 | 1,15 ± 0,3 | 0,95 ± 0,1 |
| CLDN14 | 6,39 ± 3,64 | 3,06 ± 1,4 | 4,5 ± 2,51 | 3,78 ± 2,32 |
| CLDN25 | 2,08 ± 1,41 | 0,86 ± 0,35 | 1,26 ± 0,72 | 0,88 ± 0,61 |
| CTNNB1 | 1,3 ± 0,9 | 0,69 ± 0,38 | 0,94 ± 0,48 | 0,67 ± 0,48 |
| FBN1 | 1,2 ± 0,56 | 1,04 ± 0,32 | 1,31 ± 0,36 | 0,92 ± 0,39 |
| GAPDH | 0,72 ± 0,19 | 0,93 ± 0,2 | 0,99 ± 0,07 | 0,89 ± 0,15 |
| ICAM1 | 1,22 ± 0,5 | 1,2 ± 0,4 | 1,53 ± 0,15 ** | 0,8 ± 0,32 |
| INSR | 0,75 ± 0,13 ** | 0,46 ± 0,09 | 0,62 ± 0,03 ** | 0,36 ± 0,1 |
| JAM1 | 1,12 ± 0,12 ** | 0,73 ± 0,13 | 1,01 ± 0,09 *** | 0,69 ± 0,04 |
| JAM3 | 0,85 ± 0,25 | 0,81 ± 0,19 | 0,96 ± 0,22 | 0,7 ± 0,18 |
| LRP1 | 0,82 ± 0,23 * | 0,42 ± 0,07 | 0,74 ± 0,07 *** | 0,42 ± 0,11 |
| LSR | 1,8 ± 0,85 | 1,28 ± 0,39 | 1,46 ± 0,52 | 0,94 ± 0,36 |
| MARVELD3 | 0,97 ± 0,24 | 0,75 ± 0,21 | 0,85 ± 0,18 | 0,64 ± 0,1 |
| MCAM | 0,98 ± 0,52 | 0,65 ± 0,28 | 0,94 ± 0,36 | 0,55 ± 0,32 |
| MFSD2A | 2,12 ± 1,58 | 1,08 ± 0,49 | 1,45 ± 0,59 | 0,96 ± 0,53 |
| OCLN | 1,82 ± 0,28 *** | 0,94 ± 0,07 | 1,4 ± 0,44 * | 0,7 ± 0,19 |
| PECAM1 | 2,14 ± 1,41 | 0,9 ± 0,46 | 1,35 ± 0,83 | 0,86 ± 0,63 |
| PPIA | 1,71 ± 0,66 | 1,3 ± 0,52 | 1,2 ± 0,45 | 1,47 ± 0,46 |
| RXRB | 1,76 ± 1,22 | 0,64 ± 0,28 | 1,26 ± 0,39 * | 0,56 ± 0,33 |
| SLC2A1 | 0,85 ± 0,18 **** | 2,35 ± 0,4 | 0,73 ± 0,11 **** | 2,11 ± 0,3 |
| SLC7A1 | 1,42 ± 0,14 *** | 0,93 ± 0,12 | 1,19 ± 0,16 ** | 0,84 ± 0,08 |
| SLC7A5 | 1,76 ± 0,29 * | 1,26 ± 0,2 | 2,09 ± 0,22 ** | 1,44 ± 0,21 |
| SLC16A1 | 1,61 ± 0,28 | 1,16 ± 0,24 | 1,49 ± 0,28 ** | 0,96 ± 0,1 |
| TFRC | 0,76 ± 0,26 | 0,44 ± 0,12 | 0,7 ± 0,12 ** | 0,38 ± 0,12 |
| TJP1 | 75,72 ± 119,2 | 3,67 ± 5,37 | 119,17 ± 116,88 | 48,22 ± 73,96 |
| VCAM1 | 10,76 ± 10,63 | 4,42 ± 3,42 | 4,55 ± 2,72 | 4,3 ± 2,86 |
| VEGFA | 1,31 ± 0,27 ** | 2,33 ± 0,49 | 1,11 ± 0,15 ** | 1,99 ± 0,45 |
| VIM | 2,48 ± 1,83 | 1,11 ± 0,58 | 1,07 ± 0,57 | 1,08 ± 0,84 |
| VWF | 4,39 ± 1,47 ** | 1,34 ± 0,43 | 3,09 ± 0,83 ** | 1,22 ± 0,47 |

Table S3: X-fold changes in the gene expression of housekeeping and target markers from 1321N1 cells after 5h OGD followed by 19h normoxia conditions using high-throughput qPCR barrier chip. Threshold cycle (Ct) values were normalised to the geometric mean of the Cq values of four housekeeping genes (ACTB, B2M, GAPDH and PPIA). Relative gene expression levels (x-fold values) were calculated by normalising the log2-fold changes to normoxia, mono-culture samples. Data were analysed separately for each single target using one-way analysis of variance (ANOVA), followed by Holm-Šidak’s post-hoc tests. Data are presented as mean ± SD, n=6, N=3. CLDN8, and CLDN10: n.d. all in all samples or weak expression. Statistical significance: between normoxia and OGD: *, p<0.05; **, p<0.01; ***, p<0.001, ****, p<0.0001.

| Genes\Treatments | Co-culture 1321N1, Normoxia, 5+19h | Co-culture 1321N1, OGD, 5+19h |
| --- | --- | --- |
| ABCB1 | 0 ± 0 | 0 ± 0 |
| ABCG2 | 1 ± 0,45 | 0,72 ± 0 |
| ACTB | 1 ± 0,1 | 1,09 ± 0,2 |
| AGER | 1 ± 0,36 | 0,6 ± 0,27 |
| APOE | 1 ± 0,8 | 1,18 ± 0,45 |
| B2M | 1 ± 0,16 | 0,89 ± 0,13 |
| CDH5 | 1 ± 0,42 | 0,44 ± 0,1 |
| CLDN1 | 1 ± 0,2 | 12,6 ± 8,31 |
| CLDN3 | 1 ± 0,48 | 0,41 ± 0,07 |
| CLDN4 | 1 ± 0,42 | 0,56 ± 0,06 |
| CLDN5 | 1 ± 0,82 | 0,47 ± 0,37 |
| CLDN6 | 1 ± 0,61 | 0,47 ± 0,15 |
| CLDN7 | 1 ± 0,3 | 0,96 ± 0,2 |
| CLDN8 | 1 ± 1,05 | 2,27 ± 1,92 |
| CLDN9 | 1 ± 0,42 | 0,85 ± 0,55 |
| CLDN10 | 1 ± 0,15 | 0,47 ± 0,26 * |
| CLDN11 | 1 ± 0,34 | 0,47 ± 0,18 |
| CLDN12 | 1 ± 0,34 | 0,69 ± 0 |
| CLDN14 | 1 ± 0,33 | 7,14 ± 4,31 |
| CLDN25 | 1 ± 0,89 | 1,98 ± 0,55 |
| CTNNB1 | 1 ± 0,63 | 1,58 ± 0,58 |
| FBN1 | 1 ± 0,33 | 1,53 ± 0,12 |
| GAPDH | 1 ± 0,31 | 1,24 ± 0,06 |
| ICAM1 | 1 ± 0,58 | 0,97 ± 0,37 |
| INSR | 1 ± 0,6 | 0,55 ± 0,09 |
| JAM1 | 1 ± 0,16 | 0,58 ± 0,06 * |
| JAM3 | 1 ± 0,15 | 0,87 ± 0,04 |
| LRP1 | 1 ± 0,35 | 0,79 ± 0,2 |
| LSR | 1 ± 0,2 | 0,87 ± 0,14 |
| MARVELD3 | 1 ± 0,95 | 0 ± 0 |
| MCAM | 1 ± 0,7 | 1,28 ± 0,3 |
| MFSD2A | 1 ± 0,84 | 2,17 ± 0,83 |
| OCLN | 1 ± 0,24 | 0,81 ± 0,04 |
| PECAM1 | 1 ± 0,77 | 1,13 ± 0,36 |
| PPIA | 1 ± 0,39 | 0,61 ± 0,06 |
| RXRB | 1 ± 0,85 | 1,05 ± 0,3 |
| SLC2A1 | 1 ± 0,31 | 3,31 ± 0,34 ** |
| SLC7A1 | 1 ± 0,54 | 0,96 ± 0,09 |
| SLC7A5 | 1 ± 0,23 | 1,89 ± 0,13 ** |
| SLC16A1 | 1 ± 0,33 | 1,45 ± 0,1 |
| TFRC | 1 ± 0,22 | 0,5 ± 0,09 * |
| TJP1 | 1 ± 0,37 | 1,73 ± 0,42 |
| VCAM1 | 1 ± 0,49 | 1,92 ± 0,72 |
| VEGFA | 1 ± 0,81 | 5,55 ± 0,73 ** |
| VIM | 1 ± 0,58 | 1,62 ± 0,24 |
| VWF | 1 ± 0,48 | 0,26 ± 0,17 |

Table S4: X-fold changes in the gene expression of housekeeping and target markers from hCMEC/D3 cells after 24h OGD using high-throughput qPCR barrier chip. Threshold cycle (Ct) values were normalised to the geometric mean of the Cq values of four housekeeping genes (ACTB, B2M, GAPDH and PPIA). Relative gene expression levels (x-fold values) were calculated by normalising the log2-fold changes to normoxia, mono-culture samples. Data were analysed separately for each single target using one-way analysis of variance (ANOVA), followed by Holm-Šidak’s post-hoc tests. Data are presented as mean ± SD, n=6, N=3. CLDN8, and CLDN10: n.d. all in all samples or weak expression. Statistical significance: between normoxia and OGD from the same culture type (mono/co-culture): *, p<0.05; **, p<0.01; ***, p<0.001, ****, p<0.0001; and between mono- and co-culture within the same treatment (normoxia/OGD): #, p<0.05; ##, p<0.01; ###, p<0.001; ####, p<0.0001.

| Genes\Treatments | Co-culture hCMEC/D3, Normoxia 24h | Co-culture hCMEC/D3, OGD 24h | Mono-culture hCMEC/D3, Normoxia 24h | Mono-culture hCMEC/D3,  OGD 24h |
| --- | --- | --- | --- | --- |
| ABCB1 | 1,57 ± 0,55 | 1,76 ± 0,99 | 1,57 ± 0,96 | 1,15 ± 0,55 |
| ABCG2 | 1,18 ± 0,34 | 1,05 ± 0,37 | 1,18 ± 0,36 | 1,02 ± 0,19 |
| ACTB | 1,3 ± 0,69 | 1,25 ± 0,4 | 0,93 ± 0,36 | 0,75 ± 0,08 |
| AGER | 0,94 ± 0,36 | 0,47 ± 0,25 | 0,61 ± 0,26 | 0,97 ± 1,15 |
| APOE | 0,88 ± 0,42 | 0,59 ± 0,42 | 0,69 ± 0,37 | 1,13 ± 1,31 |
| B2M | 0,91 ± 0,2 | 0,75 ± 0,11 | 1,11 ± 0,16 | 1,01 ± 0,15 |
| CDH5 | 1,66 ± 0,48 | 1,15 ± 0,46 | 1,03 ± 0,32 * | 0,56 ± 0,14 |
| CLDN1 | 1,32 ± 0,67 | 2,46 ± 1,58 | 1,51 ± 0,8 | 3,49 ± 1,32 |
| CLDN3 | 0,95 ± 0,57 | 1,86 ± 0,68 | 0,42 ± 0,32 | 1,43 ± 1,16 |
| CLDN4 | 0,97 ± 0,28 | 0,97 ± 0,33 | 0,78 ± 0,17 | 0,76 ± 0,15 |
| CLDN5 | 1,31 ± 0,44 | 0,77 ± 0,37 | 1,05 ± 0,37 * | 0,38 ± 0,15 |
| CLDN6 | 1,2 ± 0,4 | 1,05 ± 0,68 | 1,13 ± 0,3 * | 0,6 ± 0,13 |
| CLDN7 | 1,36 ± 0,53 | 1,3 ± 0,41 | 1,08 ± 0,2 | 1,16 ± 0,21 |
| *CLDN8** | 0,25 ± 0 | 0,11 ± 0,16 | 0 ± 0 | 0 ± 0 |
| CLDN9 | 0,51 ± 0,36 | 4,99 ± 7,39 | 4,48 ± 9,9 | 3,81 ± 5,97 |
| *CLDN10** | 0,76 ± 0 | 0,88 ± 0 | 1,09 ± 0,57 | 0,98 ± 0,15 |
| CLDN11 | 1,07 ± 0,21 | 0,95 ± 0,21 | 1,3 ± 0,23 * | 0,91 ± 0,12 |
| CLDN12 | 1,16 ± 0,2 | 1 ± 0,21 | 1,12 ± 0,17 | 0,86 ± 0,12 |
| CLDN14 | 1,76 ± 1,12 | 2,16 ± 1,19 | 0,78 ± 0,43 | 1,8 ± 1,01 |
| CLDN25 | 1,95 ± 1,03 | 1,04 ± 0,71 | 0,86 ± 0,56 | 1,89 ± 2,04 |
| CTNNB1 | 1,11 ± 0,48 | 0,74 ± 0,62 | 0,69 ± 0,34 | 1,11 ± 1,17 |
| FBN1 | 1,48 ± 0,66 | 0,72 ± 0,31 | 1,14 ± 0,38 | 1,13 ± 0,83 |
| GAPDH | 0,97 ± 0,17 | 0,79 ± 0,19 | 1,06 ± 0,16 | 0,86 ± 0,17 |
| ICAM1 | 1,14 ± 0,48 | 0,6 ± 0,24 | 0,97 ± 0,29 | 0,79 ± 0,43 |
| INSR | 1,23 ± 0,41 | 0,87 ± 0,41 | 0,95 ± 0,14 ** | 0,59 ± 0,09 |
| JAM1 | 1,03 ± 0,14 | 0,89 ± 0,12 | 0,9 ± 0,06 | 0,83 ± 0,18 |
| JAM3 | 1,18 ± 0,3 | 1,02 ± 0,24 | 1,24 ± 0,36 | 0,91 ± 0,15 |
| LRP1 | 1,06 ± 0,3 * | 0,55 ± 0,21 | 0,87 ± 0,13 ** | 0,43 ± 0,14 |
| LSR | 0,56 ± 0,31 | 0,6 ± 0,45 | 0,56 ± 0,14 | 0,53 ± 0,18 |
| MARVELD3 | 1,21 ± 0,37 | 1,16 ± 0,57 | 1,05 ± 0,32 | 0,78 ± 0,14 |
| MCAM | 0,95 ± 0,47 | 0,51 ± 0,25 | 0,76 ± 0,35 | 0,66 ± 0,57 |
| MFSD2A | 1,28 ± 0,64 | 0,64 ± 0,42 | 0,89 ± 0,31 | 1,2 ± 1,32 |
| OCLN | 1,22 ± 0,32 | 1,38 ± 0,2 | 0,87 ± 0,18 | 1,32 ± 0,31 |
| PECAM1 | 1,48 ± 0,8 | 0,66 ± 0,49 | 0,7 ± 0,48 | 1,15 ± 1,49 |
| PPIA | 1,04 ± 0,38 | 2,19 ± 0,82 | 0,85 ± 0,21 * | 1,89 ± 0,72 |
| RXRB | 1,27 ± 0,58 | 0,99 ± 0,68 | 0,78 ± 0,25 | 1,62 ± 1,55 |
| SLC2A1 | 1,2 ± 0,34 | 0,68 ± 0,27 | 0,85 ± 0,15 ** | 0,48 ± 0,11 |
| SLC7A1 | 1,42 ± 0,38 | 1,25 ± 0,42 | 1,07 ± 0,21 | 1,02 ± 0,33 |
| SLC7A5 | 1,23 ± 0,3 | 1,74 ± 0,66 | 0,99 ± 0,24 * | 1,71 ± 0,46 |
| SLC16A1 | 1,42 ± 0,18 | 1,42 ± 0,3 | 1,04 ± 0,18 | 1,23 ± 0,24 |
| TFRC | 1,14 ± 0,33 | 0,72 ± 0,22 | 0,88 ± 0,14 * | 0,6 ± 0,11 |
| TJP1 | 162,41 ± 124,85 | 88,04 ± 121,26 | 0,7 ± 0,3 | 171,67 ± 210,93 |
| VCAM1 | 3,58 ± 2,28 | 4,07 ± 5,95 | 0,98 ± 0,5 | 3,88 ± 2,53 |
| VEGFA | 1,52 ± 0,56 | 1,06 ± 0,45 | 1,01 ± 0,22 | 0,64 ± 0,18 |
| VIM | 1,89 ± 0,9 | 0,75 ± 0,52 | 0,85 ± 0,5 | 1,73 ± 2,36 |
| VWF | 1,25 ± 0,36 | 1,32 ± 0,47 | 0,85 ± 0,19 | 1,09 ± 0,46 |

Table S5: Median size and zeta potential of 1321N1 and hCMEC/D3 cells after sEV purification. Data are presented as mean ± SD, n=3-9.

|  | 1321N1 | hCMEC/D3 |
| --- | --- | --- |
| Median size | 145.9 ± 7.99 | 127.55 ± 4.88 |
| Zeta potential | -35.09 ± 9.21 | -41.17 ± 0.15 |

Table S6: sEV labelling efficiencies in apical-to-basolateral uptake experiments. Data are presented as mean ± SD, N=3.

| **sEV labelling efficiency (%) - Apical-to-basolateral direction** | | | |
| --- | --- | --- | --- |
| HEK293T | A549 | MDA-MB-231 | SK-MEL-28 |
| 31.4 ± 5.17 | 32.94 ± 8.092 | 39.92 ± 26.87 | 15.86 ± 4.997 |
| 1321N1 | MO3.13 | HMC3 | SH-SY5Y |
| 29.58 ± 12.15 | 23.65 ± 13.88 | 40.43 ± 12.79 | 36.66 ± 9.668 |

Table S7: sEV labelling efficiencies in basolateral-to-apical uptake experiments. Data are presented as mean ± SD, N=3.

| **sEV labelling efficiency (%) - Basolateral-to-apical direction** | | | |
| --- | --- | --- | --- |
| HEK293T | A549 | MDA-MB-231 | SK-MEL-28 |
| 31.4 ± 5.17 | 42.35 ± 15.47 | 52.87 ± 16.23 | 21.09 ± 12.88 |
| 1321N1 | MO3.13 | HMC3 | SH-SY5Y |
| 29.85 ± 7.97 | 21.43 ± 11.44 | 39.28 ± 11.38 | 54.34 ± 10.4 |

Table S8: sEV labelling efficiencies in OGD experiments. Data are presented as mean ± SD, N=3.

| **sEV labelling efficiency (%)** | | | |
| --- | --- | --- | --- |
| 5h OGD+19h Normoxia | | 24h OGD | |
| HEK293T | SH-SY5Y | HEK293T | SH-SY5Y |
| 21,77 ± 13,85 | 23,67 ± 2,93 | 13,47 ± 5,2 | 18,33 ± 3,23 |
| AP to BL | BL to AP | AP to BL | BL to AP |
